# Novel Approaches for the Recovery of Natural Pigments with Potential Health Effects

**DOI:** 10.1021/acs.jafc.1c07208

**Published:** 2022-01-18

**Authors:** Celia Carrillo, Gema Nieto, Lorena Martínez-Zamora, Gaspar Ros, Senem Kamiloglu, Paulo E. S. Munekata, Mirian Pateiro, José M. Lorenzo, Juana Fernández-López, Manuel Viuda-Martos, José Ángel Pérez-Álvarez, Francisco J. Barba

**Affiliations:** † Nutrición y Bromatología, Facultad de Ciencias, Universidad de Burgos, E-09001 Burgos, Spain; ‡ Department of Food Technology, Nutrition and Food Science, Veterinary Faculty, University of Murcia, 30100 Murcia, Spain; § Department of Food Engineering, Faculty of Agriculture, Bursa Uludag University, 16059 Gorukle, Bursa, Turkey; ∥ Science and Technology Application and Research Center (BITUAM), Bursa Uludag University, 16059 Gorukle, Bursa, Turkey; ⊥ Centro Tecnológico de la Carne de Galicia, Avenida Galicia No. 4, Parque Tecnológico de Galicia, San Cibrao das Viñas 32900, Ourense, Spain; # Área de Tecnología de los Alimentos, Facultad de Ciencias de Ourense, Universidad de Vigo, 32004 Ourense, Spain; ¶ IPOA Research Group, Agro-Food Technology Department, Centro de Investigación e Innovación Agroalimentaria y Agroambiental (CIAGRO-UMH), Miguel Hernández University, 03312 Alicante, Spain; + Nutrition and Food Science Area, Preventive Medicine and Public Health, Food Science, Toxicology and Forensic Medicine Department, Faculty of Pharmacy, Universitat de València, Avda. Vicent Andrés Estellés, s/n, 46100 Burjassot, València, Spain

**Keywords:** bioactive colorants, food byproducts, novel extraction technologies, functionality

## Abstract

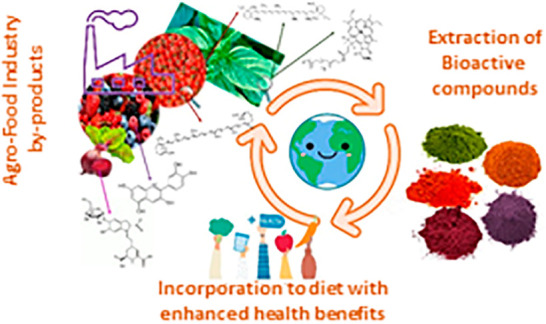

The current increased industrial food production has led to a significant rise in the amount of food waste generated. These food wastes, especially fruit and vegetable byproducts, are good sources of natural pigments, such as anthocyanins, betalains, carotenoids, and chlorophylls, with both coloring and health-related properties. Therefore, recovery of natural pigments from food wastes is important for both economic and environmental reasons. Conventional methods that are used to extract natural pigments from food wastes are time-consuming, expensive, and unsustainable. In addition, natural pigments are sensitive to high temperatures and prolonged processing times that are applied during conventional treatments. In this sense, the present review provides an elucidation of the latest research on the extraction of pigments from the agri-food industry and how their consumption may improve human health.

## Introduction

Over the past several decades, industrial-scale and centralized production systems, such as large-scale farming and food processing and distribution, have increased to meet the demand for food associated with the growing human population worldwide.^1^ Accordingly, processing of foods of plant origin including fruits and vegetables, cereal grains, herbs and spices, nuts, etc. generates large amounts of byproducts, which are often discarded as waste. Damaged raw materials, peels or skins, seeds, brans, husk, hulls, cobs, oilseed cakes, spent grains, molasses are some examples of these processing byproducts, which account for approximately 190 million tonnes per year on a global scale.^2^ For both economic and environmental reasons, the large amount of food waste produced during manufacturing has been investigated for the recovery of valuable components.^3^

Due to both their coloring properties and potential positive effects on human health, recovery of natural pigments including anthocyanins, betalains, carotenoids, and chlorophylls from food wastes is critical.^4^ Conventional solid–liquid extraction techniques used to recover natural pigments from food wastes are often time-consuming, expensive, and unsustainable.^2^ In recent years, novel extraction technologies such as pulsed electric field, ultrasound-, microwave-, and high-pressure-assisted-extraction, among others, gained importance due to the increased consumer demand for nutritious foods that are produced using environmentally friendly technologies. The advantages of the use of novel technologies to extract natural pigments from plant products include better isolation, higher selectivity, reduced energy consumption, and low environmental impact.^5^

Considering the above information, the aim of this review paper is to provide a recent update on novel extraction techniques that are utilized for the recovery of natural pigments including anthocyanins, betalains, carotenoids, and chlorophylls from food wastes. First, we presented the significant food waste sources utilized for the extraction of natural pigments. Following that, the effectiveness of novel technologies, such as pulsed electric field, ultrasound-, microwave-, and high-pressure-assisted-extraction, on the recovery of natural pigments were discussed. Finally, we highlighted the functional and bioactive properties attributed to recovered natural pigments from food wastes using novel approaches.

## Anthocyanins

### Sources of Anthocyanins

**Figure 1:**
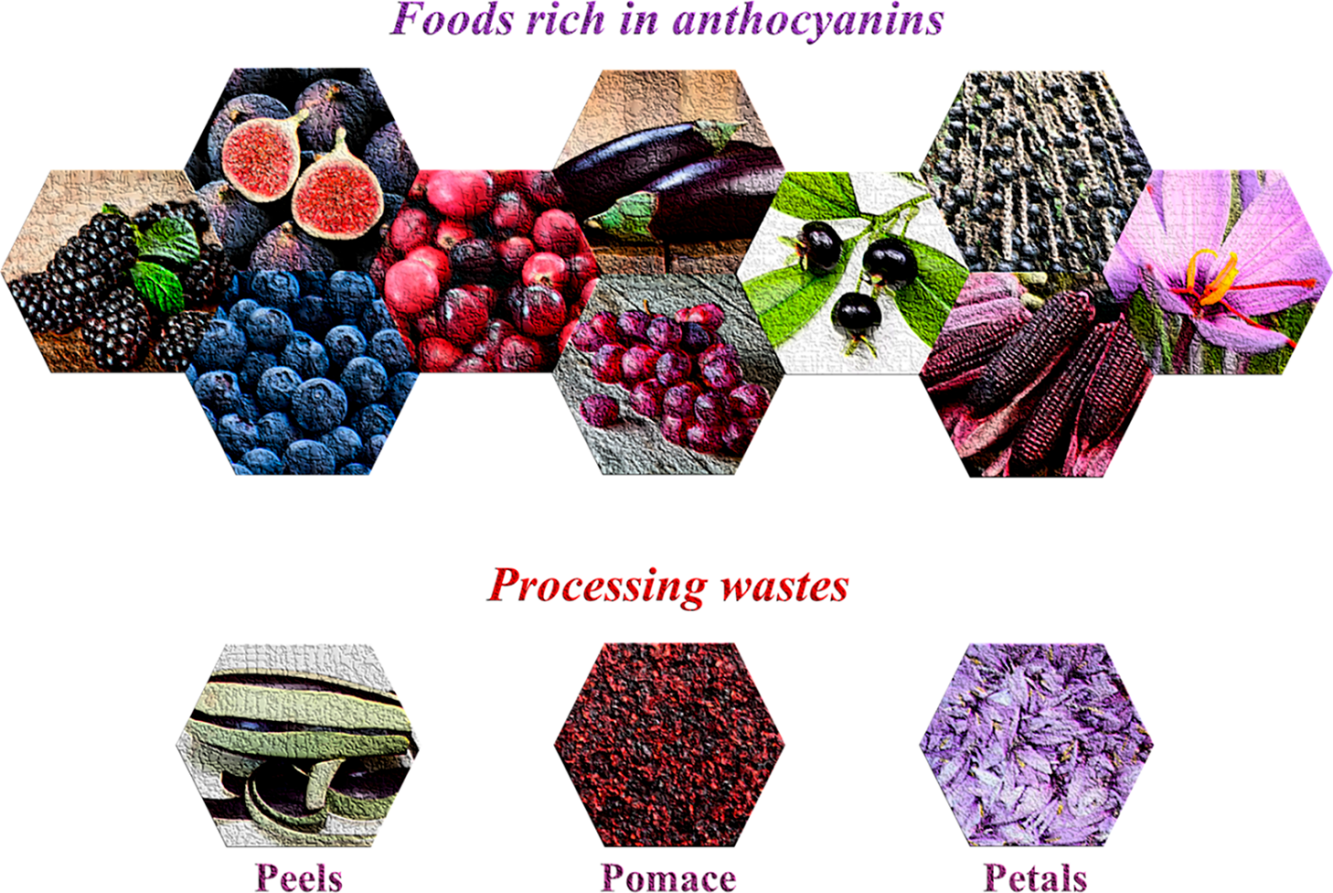
Sources of anthocyanin-rich wastes.

Anthocyanins water-soluble pigments belonging to the group of secondary metabolites within the class of phenolic compounds.^6^ They are found as glycosides of their respective aglycones, called anthocyanidins. Cyanidin, delphinidin, malvidin, pelargonidin, peonidin, and petunidin are major anthocyanidins present in foods.^7^ These compounds are widely distributed in fruit and vegetables such as blackberry (*Rubus fruticosus*), blueberry (*Vaccinium myrtillus*), cranberry (*Vaccinium macrocarpon*), fig (*Ficus carica* L.), grapes (*Vitis* sp.), grumixama (*Eugenia brasiliensis*), juçara (*Euterpe edulis* Mart.), purple corn (*Zea mays*), and petals of saffron (*Crocus sativus*) (Figure 1).

The berries group is a comprised of several small size fruits with intense color varying from purple and dark blue to black that are usually pressed for juice production. The residue generated from berries juice producing are mainly composed of peels, seeds, and stems that can reach up to 30% of the fruit weight.^8^ Grapes are fruits largely produced on all continents, wherein the main varieties produced in the past decade were Cabernet Sauvignon, Merlot, Chardonnay, and Syrah, especially for wine production.^9^ Regarding grape processing into wine, pomace (peels, seeds, and stalks) comprise between 15 and 25% of the total grape weight.^10^

Purple corn is a variety of corn with intense and characteristic purple color that has originated from South America in the Andean region. Milling purple corn into a fine flour has a processing waste generation of around 36%.^11^ Saffron production is a highly appreciated food ingredient with intense red color. The plant part commercialized as “saffron” is the daughter corm, which is obtained from drying the red colored saffron flower stigma. Saffron plant production has a limited yield, and its main production residue is comprised of flower petals that have a characteristic purple color. It is estimated that up to 300 000 flowers are necessary to produce 1 kg of saffron spice.^12^ This scenario indicates the necessity to improve the management of residues rich in anthocyanins and explore the recovery of this highly valuable compound.

### Novel Approaches for the Extraction of Anthocyanins from Food Waste

The extraction of anthocyanins from food processing residues with novel strategies has been explored in several studies in the last few years (Table 1).

**Table 1:** Extraction of Anthocyanins with Emerging Technologies

source of anthocyanins	technology	extraction conditions	extraction yield^a^	refs
Jabuticaba epicarp (*Myrciaria jaboticaba* (Vell.) Berg.)	ultrasound	power 500 W, temperature 30–25 °C, time 24.4 min, 34.5% ethanol, solvent/solid ratio 20 mL/1 g	31 mg cyanidin-3-*O*-glucoside equivalents/g extract	(13)
fig (*Ficus carica* L.)	ultrasound	power 310 W, time 21 min, 100% ethanol, solid/solvent ratio 183 g/L	3.8 mg cyanidin-3-rutinoside equivalents/g dry weight	(14)
eggplant (*Solanum melongena* L.) peel	ultrasound	frequency 45 kHz, temperature 50 °C, time 50 min, solvent 100% methanol, solvent/solid ratio 10 mL/1 g	2 275 mg/kg mg cyanindin-3-glucoside equivalents	(15)
purple corn (*Zea mays* L.) bran	ultrasound	power 400 W, time 35 min, solvent 95% ethanol, solvent/solid ratio 8:1, 90 pulses	3.6 g cyanidin-3-*O*-glucoside equivalents/kg	(16)
grumixama (*Eugenia brasiliensis*), blackberry (*Rubus fruticosus*) and blueberry (*Vaccinium myrtillus*) residues	ultrasound	power 580 W, frequency 37 kHz, temperature 80 °C, time 90 min, 50 and 70% ethanol, solvent/solid ratio 45 mL/2 g	grumixama: 0.74–0.87 mg cyanidin-3-*O*-glucoside equivalent/g dry weight, blackberry: 2.38 mg cyanidin-3-*O*-glucoside equivalent/g dry weight, blueberry: 2.07–2.33 mg cyanidin-3-*O*-glucoside equivalent/g dry weight	(17)
blackberry (*Rubus fruticosus*) residues	ultrasound	power 150 W, frequency 37 kHz, time 10 min, room temperature, absolute ethanol, solvent/solid ratio 15 g/200 mL	6.6 mg cyanidin-3-*O*-glucoside equivalent/g dry weight	(19)
juçara (*Euterpe edulis* Mart.) residue	ultrasound	power 800 W, frequency 19 kHz, time 45 min	8.7 mg cyanidin-3-rutinoside equivalent/g dry weight	(18)
wine lee	ultrasound	time 30–90 s, amplitude 10–100%, solvent 50% ethanol, temperature 25 °C, solvent/solid ratio 10 mL/1 g	2.91 mg malvidin-3-*O*-glucoside equivalents/g dry weight	(20)
				
grape pomace	pulsed electric field	field strength 1.2 kV/cm, energy input 18 kJ/kg, density 1.0 g/cm^3^, temperature 20–50 °C, solvent 50% ethanol, solvent/solid ratio 5	increased total anthocyanins to total flavan-3-ols ratio (from 7.1 to 9.0)	(21)
blueberry processing byproducts	pulsed electric field	field strength 20 kV/cm, pulses 10, solvent 60% ethanol acidified (0.1% hydrochloric acid), temperature 40 °C, solvent/solid ratio 6:1	223.13 mg cyanidin 3-glucoside equivalent/L	(22)
				
saffron (*Crocus sativus*) industry residues	supercritical CO_2_	flow rate 100 mL/min, temperature 62 °C, pressure 16.4 MPa, cosolvent 5% (99.9% ethanol), time 47 min	103.4 mg/100 g dry weight	(23)
juçara (*Euterpe edulis* Mart.) residue	supercritical CO_2_	flow rate 2.1 × 10^–4^ kg/s, pressure 20 MPa, cosolvent 10% (50% ethanol acidified, pH 2.0), temperature 60 °C	22 mg cyanidin-3-rutinoside equivalent/g dry weight	(18)
				
juçara (*Euterpe edulis* Mart.) residue	pressurized liquid extraction	flow rate 1.5 mL/min, pressure 10 MPa, temperature 40 °C, solvent acidified water (pH 2.0), solvent/solid ratio 9:1	9.2 mg cyanidin-3-rutinoside equivalent/g dry weight	(18)
blackberry (*Rubus fruticosus* L.) residues	pressurized liquid extraction	flow rate 3.35 mL/min, pressure 7.5 MPa, temperature 100 °C, solvent 50% ethanol, solvent/solid ratio 18 g/1 g	1.02 mg cyanidin-3-*O*-glucoside/g	(24)
grumixama (*Eugenia brasiliensis*), blackberry (*Rubus fruticosus*) and blueberry (*Vaccinium myrtillus*)	pressurized liquid extraction	pressure 10.0 MPa, temperature 80 °C, time 30 min, solvent 50 and 70% ethanol and acidified water, solvent/solid ratio 18 kg/1 kg	grumixama 0.20–0.44 mg cyanidin-3-*O*-glucoside equivalent/g dry weight	(17)
			blackberry 1.49–1.72 mg cyanidin-3-*O*-glucoside equivalent/g dry weight	
			blueberry 1.32–1.69 mg cyanidin-3-*O*-glucoside equivalent/g dry weight	
cranberry pomace	pressurized liquid extraction	pressure 5 MPa, temperature 60–120 °C, solvent 100% ethanol	5.22–7.78 mg cyanidin 3-glucoside equivalent/L	(25)
				
fig (*Ficus carica* L.) peel	microwave	power 400 W, time 5 min, temperature 62.4 °C, solvent 100% ethanol, solid/solvent ratio 1:20	4.11 mg cyanidin 3-rutinoside/g dry weight	(14)
sour cherry (*Prunus cerasus* L.) peels	microwave	power 500 W, time 90 s, solvent 80% ethanol	12.47 mg of cyanidin-3-glucoside/g	(26)
grape juice waste	microwave	428 W, time 2.2 min, solvent/solid ratio 18.4 mL/g	1.32 mg/g	(27)
blueberry (*Vaccinium* spp) bagasse	microwave	power 525 W, time 3 min, solid/liquid ratio 1:15	142.5 mg cyanidin-3-glucoside equivalent/100 g	(28)
wine lee	microwave	power 300 W, solid/liquid ratio 0.140 g/mL, solvent 40% ethanol, time 90 s	6.2 mg malvidin-3-*O*-glucoside equivalents/g dry weight	(20)
				
grape byproducts	ohmic heating	electric field 30 V/cm, frequency 25 kHz, temperature 100 °C, solvent 100% methanol, time 13 s	224.06 mg cyanidine-3-glucoside equivalents/kg dry weight	(29)
grape skins	ohmic heating	electric field 70 V/cm, frequency 25 kHz, temperature 100 °C, time 20 s	1.35 mg anthocyanins/g	(30)

a Extraction yield: optimum conditions.

The main technologies applied are ultrasound, pulsed electric field, supercritical CO_2_, pressurized liquid extraction, microwave, and ohmic heating. Among these technologies, ultrasound has been the most studied technology wherein several variables have been studied. For instance, a recent study reported the use of response surface methodology to evaluate and optimize the extraction time, ultrasound power, and the proportion of ethanol in the solvent.^13^ In this study, the maximum extraction yield of anthocyanins was obtained increasing the ultrasound power. Reducing the ethanol proportion in solvent was favorable to improve the extraction of anthocyanins, and a short extraction time was suitable to improve the anthocyanin content in the extract.

A similar experiment has been recently carried out to optimize the extraction of anthocyanins from figs and explored the effect of the same variables.^14^ In this case, the use of 100% ethanol solution was indicated as the optimum level of ethanol in the solvent composition along with a short extraction time and high ultrasound power to maximize the extraction of anthocyanins. Likewise, the extraction of anthocyanins from eggplant peels using ultrasound was affected by the solvent composition (especially using methanol), ultrasonic frequency, temperature, and time.^15^

A recent study explored the effect of extraction time, solid/liquid ratio, pulses, and ultrasonic power to obtain anthocyanins from purple corn bran.^16^ All these variables were indicated as significant to improve the extraction of anthocyanins with ultrasound. Another recent experiment reported the use of ultrasound to obtain anthocyanins from berry residues.^17^ In this case, the effect of solvent was evaluated, and no significant effects were indicated between acidified water and hydroethanolic solutions. Additionally, other studies indicated that anthocyanins can be obtained from juçara (*Euterpe edulis* Mart.; a berry mainly found in Brazil) residues^18^ and blackberry (*Rubus fruticosus*) residues^19^ using ultrasound as the main extraction technology.

Along with the use as an extraction technology, the use of ultrasound has also been explored as a pretreatment to increase the conventional solvent extraction. This strategy was tested in an experiment with wine lee.^20^ In this study, no significant effects were observed among treatments with different extraction times or wave amplitudes. According to the authors, the phenomenon of cavitation improved the external mass transfer, whereas internal mass transfer remained not affected by ultrasound treatment, which limited the extraction process.

Pulsed electric fields are another technology with a relevant effect in the extraction of anthocyanins from food residues. An interesting outcome for the use of this technology was reported in a study with grape pomace.^21^ The application of a pulsed electric field in the samples was associated with an increase in anthocyanin content of the obtained extract. This result was interpreted as a favorable selectiveness toward the extraction of anthocyanins by the ratio between anthocyanins and total flavan-3-ols content. This ratio was improved due to the optimization of the energy input, field strength, and density of the treated sample. Another related study highlighted the importance of optimizing the intensity of the electric field, number of applied pulses, and solvent proportion and composition to improve the extraction anthocyanins from blueberry processing byproducts.^22^

An interesting option to recover anthocyanins from food byproducts is the use of supercritical CO_2_. The use of this technology was recently tested to improve the extraction of anthocyanins from saffron residues (mainly composed of petals).^23^ Mild temperature with high pressure and carrying out the extraction in less than 1 h were important advances reported in this study. The use of this technology was also applied to recover anthocyanins from juçara residue.^18^

Pressurized liquid extraction has been suggested as an interesting option to obtain anthocyanin-rich extracts. The optimization of this technology was recently tested in a juçara residue, and the optimum conditions were active with mild heating treatment and the use of acidified water as a solvent.^18^ Conversely, the use of this technology in blackberry residues revealed that hydroethanolic solution and heating up to 100 °C was necessary to maximize the recovery of anthocyanins.^24^ Another experiment from the same research group explored the use of this technology in three berries: grumixama, blackberry, and blueberry.^17^ In this case, nonsignificant differences were obtained from changes in the solvent composition (50 and 70% ethanol and acidified water). An experiment with cranberry pomace highlighted the importance of optimizing pressure and solvent composition to increase the extraction of anthocyanins from cranberry pomace.^25^ However, temperature had a nonsignificant effect in the range of 40–120 °C.

Microwaves can greatly assist in the recovery of anthocyanin from different food wastes. Recent experiments have been focusing on process optimization with response surface methodology, wherein the short extraction time is a common characteristic of this technology. An example of the improvement associated with microwave-assisted extraction was reported in the recovery of anthocyanins from fig peels.^14^ In this residue, the optimum condition was achieved with a mild heating of the sample and the use of pure ethanol as the extracting solvent. A related experiment with sour cherry peel indicated that increasing microwave power, irradiation time, and the proportion of ethanol in solvent (500 W, 90 s, and 80% ethanol, respectively) were favorable aspects to recover anthocyanins.^26^

The use of microwave heating was also evaluated to obtain anthocyanins from grape waste, wherein the optimum extraction conditions were obtained with 428 W, 2.2 min of irradiation, and using a solvent/solid ratio of 18.4 mL/g.^27^ Conversely, a recent experiment with blueberry bagasse explored the effect of temperature and power in the extraction of anthocyanins.^28^ A not significant improvement in the anthocyanin content in the obtained extract in relation to extraction with conventional solvent extraction method were reported. Alternatively, microwave heating was also tested as a pretreatment to conventional solvent extraction.^20^ In this case, the optimization was associated with a significant increase in anthocyanins content in relation to conventional solvent extraction without any pretreatment.

Another interesting technology to improve the recovery of anthocyanins from residual sources is ohmic heating. Recent studies have explored its use as a pretreatment to conventional solvent extraction. For instance, ohmic heating favored the release of anthocyanins from grape pomace with an important reduction in extraction time (from 15 min to few seconds) and also prevented the degradation obtained from application of higher temperatures for long periods.^29^ However, no significant increase in anthocyanin extraction yield was reported in relation to conventional solvent extraction at room temperature. A similar experiment with ohmic heating (as pretreatment) in grape skin indicated significant increase in the extraction of anthocyanin using a short-time high-temperature.^30^ This study also indicated that mild ohmic heating with prolonged exposure time did not improve the release of anthocyanin in comparison to untreated residue.

Besides the methods previously described, fermentative processes have been also considered potential alternatives to assist in the extraction of bioactive compounds from food wastes. In this sense, Amaya-Chantaca et al.^31^ explored the use of grape pomace as a fermentation substrate for the extraction of phenolic compounds. In this study, two fermentation methods were compared (submerged and solid-state fermentation), and *Aspergillus niger* GH1 was used as a biological model. The results showed that both fermentation systems increased the release of phenolic compounds, although the highest yields were observed when using solid-state fermentation. Anthocyanins were among the main compounds identified in the extracts. An increase in the recovery of anthocyanins by solid-state bioprocessing has been also observed in chokeberry pomace fermented with *Aspergillus niger* and *Rhizopus oligosporus*.^32^ These findings highlight this method as a potential alternative for the extraction of these red pigments.

In general, novel extraction favor the release of anthocyanins from different food wastes, wherein advantages in terms of extraction time and compatibility with food grade solvents (especially acidified water and hydroethanolic mixtures) can be seen as favorable aspects for further application in functional food development. It is also important to remember that some recommendations to improve the anthocyanin extraction yield in the conventional solvent method such as low temperature and acidified water may not play a major role. This consideration is supported by the diversity of mechanisms and effect attributed to each technology, such as cavitation in ultrasound and electroporation in a pulsed electric field. However, further experiments are necessary to clarify optimum conditions and the role of the matrix and avoid a low extraction yield.

### Influence of Novel Extraction Technologies on the Bioactive Properties of Anthocyanins

Anthocyanins have been associated with many potential health benefits. Considering the importance of novel extraction methods in the extraction of anthocyanins, recent studies have characterized the effect of these methods in the biological potential of extracted anthocyanins. The antioxidant activity *in vitro* has been the most studied biological effect explored in recent studies.

Ultrasound extraction seems to not favor the antioxidant activity of anthocyanin extracts. This outcome was reported in a recent study with eggplant where solvent (methanol), temperature (60 °C), and time (20 min) were associated with improved antioxidant activity in the obtained extract.^15^ However, the optimum ultrasound frequency was 0 kHz. A related experiment with wine lee indicated that ultrasound extraction did not affect antioxidant activity of extracted compounds.^20^ Another study with ultrasound treatment in different berries (blackberry, blueberry, and grumixama) indicated that significant reductions in antioxidant activity of extracts were obtained in relation to conventional solvent extraction, regardless of solvent composition.^17^

In the case of pressurized liquid extraction, contrasting results have been reported in recent experiments. The use of pressurized liquid extraction in blackberry caused significant increases in antioxidant activity evaluated with DPPH and ORAC assays.^24^ A similar outcome was reported for the extraction of antioxidant compounds from juçara using acidified 50% ethanol.^18^ The DPPH assay revealed that a significant increase in antioxidant activity was obtained by increasing the temperature. Regarding the ORAC, the highest mean values were obtained using this solvent an no significant effect was indicated in relation to temperature (40–80 °C).

Solvent is another relevant factor to consider when using pressurized liquid extraction to obtain antioxidant compounds. This consideration was related to a recent experiment with cranberry residue where the relation between antioxidant activity (FRAP assay) and anthocyanin content was influenced by solvent composition.^25^ When acidified water was used as the solvent, antioxidant activity and anthocyanin content displayed a negative correlation. Conversely, the use of ethanol produced a positive correlation between antioxidant activity and anthocyanin content. Although these studies indicate a positive effect of pressurized liquid extraction in the recovery of anthocyanins and antioxidant activity in the extracts, a related study with differences indicated that this novel technology did not affect the antioxidant activity of the anthocyanin-rich extract in relation to conventional solvent extraction.^17^

Microwave heating is another novel technology that has been associated with the production of antioxidant extracts. This outcome was reported by an experiment with wine lee.^20^ Both DPPH and ORAC assay indicated that microwave pretreatment improved the antioxidant activity of the extract in relation to conventional solvent extraction. Another experiment with this technology indicated a positive correlation between total and anthocyanin contents and antioxidant activity measured with the DPPH assay in a cherry residue extract.^26^

In the case of supercritical CO_2_ extraction, the extraction carried out in optimum conditions (at 62 °C and 16.4 MPa with 5% cosolvent (99.9% ethanol) in supercritical CO_2_ for 47 min) increased the antioxidant activity of jaboticaba extract (rich on anthocyanins) measured by DPPH and FRAP assay.^23^

Ohmic heating pretreatment increased DPPH radical scavenge activity in relation to conventional solvent extraction when methanol with 1% HCl, lactic acid, or citric acid solvents were used to obtain an anthocyanin-rich extract from grape byproducts.^29^ However, no significant difference was reported when water was used as the solvent. The evaluation of antioxidant activity by the ORAC assay also revealed that this novel extraction method increased the antioxidant activity in the extract obtained with citric acid in relation to conventional solvent extraction. Other solvents led to no effect (water and lactic acid) or significant reduction (methanol with 1% HCl) in antioxidant activity. It is worth commenting that this study also explored the antimicrobial effect of the extract obtained with ohmic heating against pathogenic bacteria *Bacillus cereus*, *Escherichia coli*, *Pseudomonas aeruginosa*, *Salmonella enteritidis*, methicillin-resistant and -sensitive *Staphylococcus aureus* strains, and *Yersinia enterocolitica*. The most intense inhibitory effect *in vitro* was obtained with citric acid as the solvent, whereas the other solvents did not inhibit microbial growth.

These studies indicate that some technologies (particularly microwave heating, supercritical CO_2_, and ohmic heating) can be explored to improve both anthocyanin and antioxidant activity of extracts obtained from food wastes. In terms of antimicrobial activity, major efforts are still necessary to clarify the effect of these extracts.

## Betalains

### Sources of Betalains

Betalains are water-soluble nitrogen pigments responsible for the color of a limited family of vegetables. These pigments are composed of a nitrogenous core of betalamic acid [4-(2-oxoethylidene)-1,2,3,4-tetrahydropyridine-2,6-dicarboxylic acid]. Betalamic acid can either condense with amines to form betaxanthins (yellow pigments) or with imino compounds (cyclo-DOPA and/or its derivatives) to form betacyanins (violet pigments).^33^

These compounds can be mainly found in vegetables such as beetroot (*Beta vulgaris*), prickly pear (*Opuntia* sp.), pitaya fruit (*Hylocereus* sp.), and amaranth (*Amaranthus tricolour*).^34^ The different colored phenotypes of these vegetables are related to their relative content of betacyanins and betaxanthins.

Beetroot is one of the richest sources of betalains in nature, with values ranging from 3 754 to 11 932 (mg/kg dry weight), depending on the species, cultivar, or growing conditions.^35,36^ Europe is the main producer of beetroot (around 70% of the global production), and the United Kingdom generates alone huge amounts of wastes as a consequence of the outstanding beetroot juice production that occurs in this country.^37^ Taking into account the health-related properties attributed to beetroot products^38−40^ and the subsequent increasing popularity of this vegetable, the market of beetroot is expected to increase significantly during the next decade. These data highlight the need of valorization approaches to reduce the generation of wastes. In particular, industrial beetroot processing generates large amounts of pulp waste (mainly derived from the juice industry), along with peels, pomace, leaves (which represent 50% of the whole plant), and stalks, which are generally discarded after the processing of these vegetables.^41^ These unexploited byproducts contain considerable amounts of pigments and are therefore worthwhile to be recovered and used as ingredients for functional foods.

Prickly pear or cactus pear fruits are mainly produced in Mexico (44% of the global production, with 100 866 tonnes per year)^42^ and are available in a wide variety of colors (red, violet, green, or yellow), depending on the genetic origin. These fruits can be processed into different products such as juices, jams, or candies, and during their processing, large amounts of byproducts are generated, mainly peels (which account for 40–50% of the whole fruit) and pulps, which are both good sources of betalains.^42^

Pitaya or dragon fruits are exotic fruits that can be distinguished based on the color of their peel and pulp (red peel-white pulp; red peel-red pulp; yellow peel-white pulp).^43^ Amaranth is a leafy vegetable found in the south of Asia and widely consumed in Bangladesh, India, and several African countries, both cooked and in salads. Their byproducts are also interesting raw materials for the food industry as natural pigment sources.^44^ In view of the above information, it is important to investigate the recovery of these high-added value pigments through modern sustainable technologies.

### Novel Approaches for the Extraction of Betalains from Food Waste

So far, several studies have investigated novel extraction approaches for the recovery of betalains from food residues (Table 2).

**Table 2:** Extraction of Betalains with Emerging Technologies

source of anthocyanins	technology	extraction conditions	extraction yield^a^	ref
red beet stalks	ultrasound	temperature 53 °C, power 89 w, time 35 min, solid/liquid ratio 1 g powder/19 mL, solvent water	1.28 mg betacyanin/g	(45)
			5.31 mg betaxanthin/g	
beet leaves	ultrasound	power 90 W, solid/solvent ratio 1:20, time 16 min, solvent water	949.1 μg betaxanthin/g dry weight	(46)
			562.2 μg betacyanin/g dry weight	
red beetroot waste	ultrasound	frequency 44 kHz, time 30 min, temperature 30 °C, solid/solvent ratio 1 g dried powder/25 mL, solvent 30% ethanol	3 mg total betalain/g dry weight (ratio betacyanin/betaxanthin 1)	(48)
red beet peels	ultrasound	power 200 W, frequency 37 kHz, time 30 min, solid/solvent ratio 1:20, solvent water	3.87 mg betacyanin/g dry weight	(47)
			8.61 mg betaxanthin/g dry weight	
prickly pear (*Opuntia engelmannii*) peels	ultrasound	frequency 40 kHz, 200 rpm stirring, time 1.5 min, solid/liquid ratio 5 g powder/L, 50% methanol, temperature 20 °C	197.51 mg/g extract	(49)
red prickly pear peel and pulp	ultrasound	pretreatment: 10 min, 400 W, 24 kHz, 3 g fresh sample/30 mL, solvent water	89.29 mg colorants/100 g fresh weight (peels); 28.25 mg colorants/100 g fresh weight (pulps)	(50)
				
red beet peels	microwave	temperature 50 °C, time 5 min. solid/solvent ratio 1:20, solvent water	3.08 mg betacyanin/g dry sample	(47)
			1.74 mg betaxanthin/g dry sample	
white-fleshed red pitaya peels (*Hylocereus undatus*)	microwave	power 600 W, temperature 49.33 °C, time 5 min, solid/solvent ratio 1/150 g/mL, solvent water	1.51 mg betacyanins/g dry extract	(51)
prickly pear (*Opuntia engelmannii*) peels	microwave	power 400 W, temperature 25 °C, time 8.8 min, solid solvent ratio 20.3 g/L, solvent 54.8% methanol	144.6 mg betacyanins/g extract	(49)
unsalable *Amaranthus tricolor* leaves	microwave	power 200 W, temperature 31.45 °C, time 15 min, solid dried/liquid ratio 1:80 solvent water	63.3 mg betacyanin/g	(44)
			43.4 mg betaxanthin/g	
opuntia joconostle endocarp	microwave	pretreatment: 297 W, 5.5 min. extraction: temperature 5 °C, time 10 min, solvent water	8.47 mg betanin/100 g	(53)
				
red prickly pear peel and pulp	pulse electric field	pretreatment: 50 pulses at 20 kV/cm, solvent water, solid/liquid ratio 3 g fresh sample/30 mL	81.3 mg colorants/100 g fresh weight (peels); 34.25 mg colorants/100 g fresh weight (pulps)	(50)
				
pitaya fruits peels (PFP), red beet stalks (RBS), cactus pear peels (CPP)	pressurized hot water extraction	PFP: 56.9 °C, 6.7 MPa, 9 min; solid/liquid ratio 1 g/6 mL	PFP: 2.18 mg betanin equivalents (BE)/g dry extract	(54)
		RBS: 89.7 °C, 10.2 MPa, 6.5 min; solid/liquid ratio 1 g/9.9 mL	RBS: 15.35 mg BE/g dry extract	
		CPP: 70.1 °C, 9.2 MPa, 7.5 min; solid/liquid ratio 1 g/10.9 mL	CPP: 11.85 mg BE/g dry extract	

a Extraction yield: optimum conditions.

Ultrasound has been successfully applied in different beetroot byproducts. For instance, Maran and Priya^45^ carried out a study to optimize an aqueous ultrasound-assisted betalain extraction from waste red beet stalks evaluating the effect of four variables: ultrasonic power (60–120 W), extraction temperature (40–60 °C), solid–liquid ratio (1:10–1:30 g/mL), and extraction time (15–45 min). The maximum extraction yield was obtained by increasing temperature (until 55 °C), increasing ultrasonic power (until 100 W), increasing time (until 38 min), and increasing the solid/liquid ratio (until 1:25 g/mL). A similar study has been recently developed by Nutter et al.^46^ to optimize the extraction of betalains from underutilized beet leaves. These authors also explored the effect of ultrasonic power (10–90 W), solid/liquid ratio (1:20–3:20), and extraction time (4–16 min). Similarly, pigment extraction was also influenced by the variables tested. Higher betacyanin and betaxanthin yields were reached when increasing extraction time and ultrasonic power, although lower solid/liquid ratios enhanced the recoveries, which is in accordance with the ratios suggested by Maran and Priya.^45^ These authors monitored the temperature profile along the experiment and highlighted the beneficial effect that the heat generated in the medium could exert to improve the extraction yields (after 16 min of ultrasound at 100 W, the temperature in the medium increased above 80 °C). The optimized conditions suggested in this study achieved betalain yields by approximately 4.5 times higher than those obtained by conventional maceration extractions during 30 min. Seremet et al.^47^ evaluated the efficiency of an aqueous ultrasound-assisted extraction to recover betalains from red beetroot peels. These authors used an ultrasonic bath (200 W) and only evaluated the effect of the extraction time (30 vs 60 min). The extraction yield of betaxanthins was significantly higher at shorter times (30 min), although the recovery of betacyanins was not affected by the time interval. The ultrasound-assisted extraction applied in this study improved the extraction yields reached by conventional maceration extraction (48 h extraction at room temperature) by approximately 4.5 and 2 times for betacyanins and betaxanthins, respectively. An ultrasonic bath under similar conditions was used in another study for the extraction of betalains from red beetroot waste derived from the juicing industry,^48^ although these authors tested the effect of solvent on the extraction yield and highlighted 30% ethanol as the best extraction solvent to maximize the recovery of the pigments. Besides beetroot byproducts, prickly pear peels have been also valorized using ultrasound.^49^ In this study, the effect of extraction time (0.5–2.5 min), solid/solvent ratio (5–45 g/L), methanol concentration (0–100%), and temperature (3–35 °C) was evaluated, and the authors found that the solid/liquid ratio and solvent concentration were the most influential variables for betacyanins extraction (low solid/liquid ratios and methanolic concentrations increased the extraction yields).

Ultrasound have been also used as pretreatment methods to improve conventional extraction yields. In this respect, Koubaa et al.^50^ tested this approach for the recovery of betacyanins from red prickly pear peels and pulps and found that ultrasound pretreatment significantly increased pigment extraction in the case of peels (more than 2 times of an increase).

Another interesting approach for the recovery of betalains is microwave-assisted extraction, which has been recently tested in a wide variety of food wastes. For instance, Ferreres at al.^51^ conducted a study in white-fleshed red pitaya fruit byproducts and optimized different variables such as a solid/solvent ratio (1:50–1:150 g/mL), temperature (25–75 °C), and extraction time (5–65 min) using water as the extraction solvent. The results highlighted the effect of time and temperature, where short times and low/moderate temperatures maximized the extraction of betacyanins (which is in agreement with the decomposition of betacyanins into yellow degradation products, such as cyclo-dopa-5-*O*-glucoside and betalamic acid, when prolonged thermal treatments at high temperatures are applied). Sharma et al.^44^ optimized an aqueous microwave-assisted extraction to recover betalains from unsalable *Amaranthus tricolor* leaves, and besides temperature (30–90 °C) and extraction time (5–15 min), these authors also tested the effect of the microwave power (200–700 W). Lower microwave power and higher times increased the extraction of betacyanins and betaxanthins. However, while high temperatures enhanced betacyanins recovery, low temperatures were optimal in the case of betaxanthins, which is in agreement with the different stabilities attributed to these two pigments.^52^ Under the optimized conditions, microwave-assisted extraction reached significantly higher pigments extraction than a conventional Soxhlet method (15 h, 90 °C). Melgar et al.^49^ optimized similar variables (extraction time (2.5–12.5 min), solid/solvent ratio (5–45 g/L), and temperature (25–105 °C)) for the recovery of betalains from prickly pear peels, but these authors also studied the effect of the extraction solvent (methanol concentration (0–100%)). Increases in methanol concentration and extraction temperature negatively affected the extraction yields, since the effect of water polarity favors betalain extraction and thermal treatment could cause pigments losses. Red beetroot peels have been also valorized through an aqueous microwave-assisted extraction with good betacyanin recoveries (3 times higher yields than conventional maceration extraction). In contrast, the recoveries of betaxanthins were below those reached by conventional methods.^47^ Using microwaves as a pretreatment method have been also successfully applied for the recovery of betalains from xoconostle byproducts.^53^

Pulse electric fields is another technique with promising results for the extraction of betalains. This method has been tested as a pretreatment for the recovery of betacyanins from red prickly pear peels and pulps.^50^ PEF-pretreatment significantly increased the extraction yields and allowed the reduction of the extraction time in comparison with ultrasound-extraction pretreatment. In addition, the results of this study highlighted that PEF consumed less energy than ultrasound (27 kJ/kg and 800 kJ/kg for PEF and ultrasound, respectively) and is therefore more economical.

Another interesting option to recover betalains from food byproducts is the use of pressurized water extraction. This technology has been recently applied for the extraction of betalains from pitaya fruits peels, red beet stalks, and cactus pear peels.^54^ These authors demonstrated that temperature plays a key role in the extraction of betacyanins through pressurized hot water extraction, although the effect of such a variable depends on the matrix. While optimal betalain recoveries were achieved at low temperature in the case of pitaya fruit peels, maximal yields were reached at relatively high temperatures for red beet stalks. Although betacyanins are easily decomposed into cyclo-dopa-5-*O*-glucoside and betalamic acid when submitted to high thermal treatments,^52^ it is important to point out that the optimal extraction time was relatively short (9 min). Therefore, it could be hypothesized that within this short time interval, high pressure forces the water to penetrate into the pores of the food matrix and facilitates the extraction efficiency, which may compensate the degradation of the target pigments along the extraction.

Considering the above information, novel extraction technologies have demonstrated promising results for the recovery of betalains from food wastes. In general, moderate temperatures, short extraction times, and lower solid/liquid ratios are good tips to maximize the extraction of these pigments. It is important to highlight that most of the studies optimized the procedure using water as the extraction solvent, which reinforces the potential of these green approaches. However, further research is needed to clarify the role of matrix in the extraction procedure.

### Influence of Novel Extraction Technologies on the Bioactive Properties of Betalains

Betalains have been associated with several biological activities such as anti-inflammatory, antiproliferative, and antimicrobial activities, together with free radical scavenging potential, DNA-damage inhibition, gene regulation, or the prevention of lipid peroxidation.^36,55−58^ Furthermore, *in vivo* studies indicate that betalain supplementations could play a beneficial role in dyslipidemia-, oxidative stress, and inflammation-related diseases (e.g., hypertension, cancer, dyslipidemia, or stenosis of the arteries, among others). Moreover, beetroot betalains have been demonstrated to enhance exercise performance independently of nitrate physiological effects.^59−62^

However, taking into account the promising role of novel extraction technologies in the recovery of betalains, it is important to understand the impact of these methods in the functionality of the compounds recovered. In this respect, betalain rich-extracts obtained from ultrasound-assisted technologies have exhibited good antioxidant potential. This outcome has been demonstrated using beetroot wastes^48^ and cactus pear peels as extraction raw materials.^49^

Melgar et al.^49^ pointed out that the extracts obtained from cactus pear byproducts showed higher antioxidant capacity when using high concentration of water in the extraction solvent, which is in agreement with the extraction conditions required to maximize the recovery of pigments discussed in the previous section. However, when compared with conventional extraction methods, contrasting results have been obtained. Red beet peel extracts obtained by ultrasound-assisted extraction showed lower antioxidant potential (measured by DPPH and ABTS assays) than those obtained through conventional maceration extraction.^47^ In contrast, both DPPH antioxidant activity and FRAP reducing power were higher in ultrasound-derived extracts than after a Soxhlet extraction of amaranth leaves.^44^

In the case of microwave-assisted extraction, inconsistent results have been also found when comparing the antioxidant capacity of the extracts obtained through modern and conventional methods.^44,47^ Similarly, the fact of using a solvent with high aqueous concentration has demonstrated a positive effect in the antioxidant properties of the extract^49^ obtained by microwave-assisted extraction. The use of microwaves as a pretreatment method increased the antioxidant capacity of the extracts compared with nonpretreated samples.^53^

Extracts obtained by pressurized hot water extraction method have also proven to have good bioactivities.^54^ For instance, red beet stalks and cactus pear peels extracts obtained by pressurized hot water extraction exhibited good superoxide anion scavenging potential. Furthermore, these extracts could inhibit the steatosis in a cellular model as well as the intracellular reactive oxygen species. Also, genes involved in lipogenesis and lipid oxidation can be regulated by such betalain-rich extracts.^54^

These studies suggest promising health-related properties of the extracts obtained by modern extraction technologies, although further research is needed to clarify some of the bioactivities (since most of the research is focused on the study of the *in vitro* antioxidant activity) and to maximize the functionality of the extracts recovered.

## Carotenoids

### Sources of Carotenoids

Carotenoids are organic pigments mainly produced by plants and algae, and they are responsible for the characteristic yellowish, orange, and reddish color of fruits and vegetables. Furthermore, due to its solubility in fat, carotenoids are highly bioavailable in oils and fatty tissues of animals, where they are accumulated.^63^ Such is the case of salmon, flamingos, lobsters, mullets, crabs, and shellfish, among others.^63^

**Figure 2:**
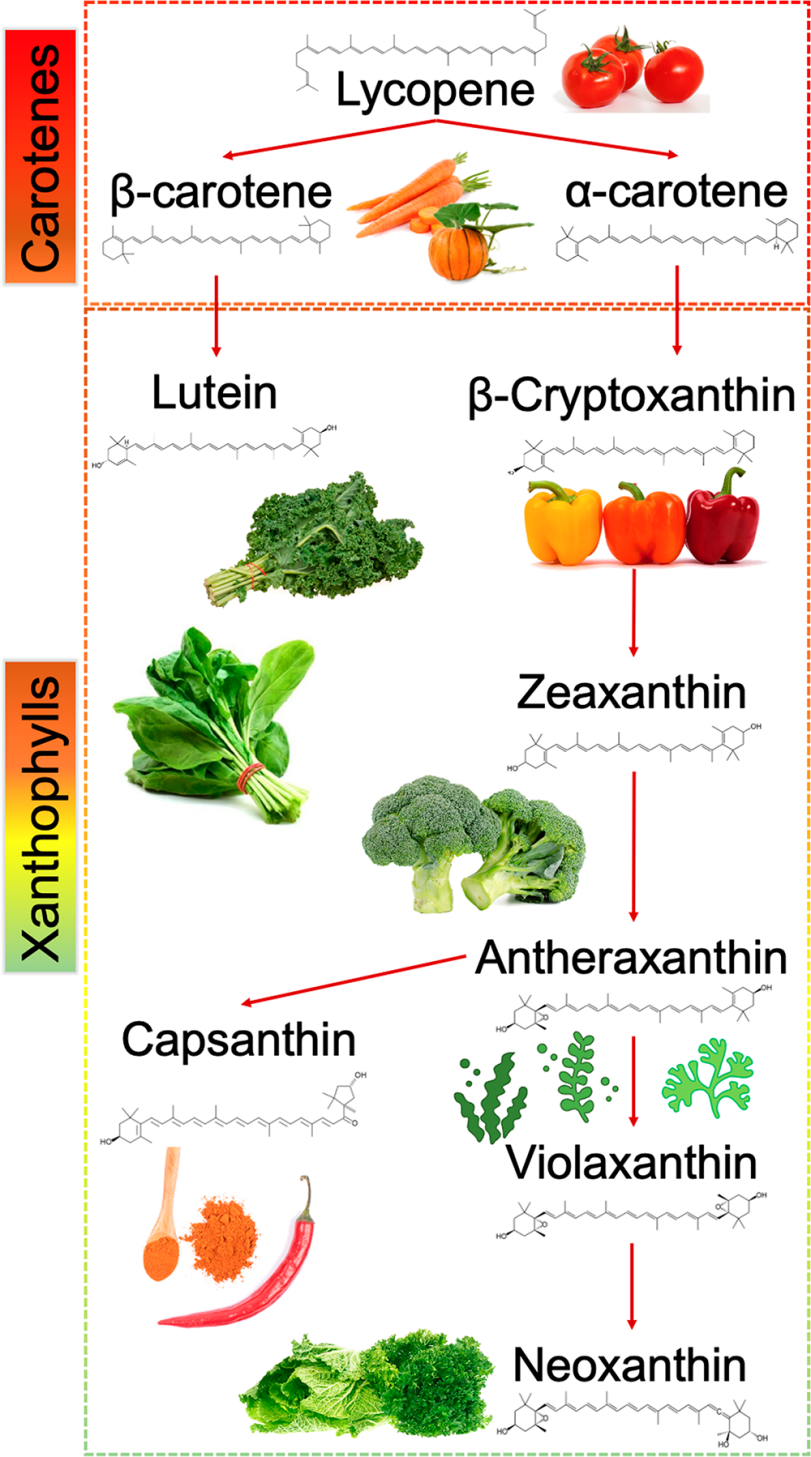
Biosynthesis and main sources of carotenoids.

More than a thousand types of carotenoids have been identified and categorized into two main groups: xanthophylls (with oxygen in its chemical formula) and carotenes (hydrocarbon chain with no oxygen in its chemical formula), and most of them have their origin in the *plantae* kingdom. In this sense, the general biochemical structure of the carotenoids is a polyene chain of double bonds and possible rings in the extremes (Figure 2), which allow their biological activities, especially their ability as an electron donor throughout the molecule, which contributes to their antioxidant activity. As a matter of fact, when plant carotenoids act as light absorbers, they help to trigger photosynthesis reactions, provide photoprotection to abiotic stressors, produce plant coloration, and stimulate cell signaling.^64,65^

Furthermore, carotenogenesis of carotenes and xanthophylls in the *plantae* kingdom is controlled by the transcript genes, which are mainly regulated by light and temperature.^66^ As the initial precursor of lycopene in the top of the carotenoid’s chain (Figure 2), phytoene synthase (PSY) plays an essential role in the conversion of geranylgeranyl diphosphate to phytoene, whose stimulation directly depends on light stimuli,^64^ especially wavelengths from 400 to 500 nm (blue and green lighting) and from 650 to 750 nm (red and far red lighting).^64,67^ As a matter of fact, this effects of blue, red, and far red led light on caretonegenesis have been recently shown in carrot sprouts^68^ and red bell peppers.^69^ On this point, transcript genes play an essential role in the production of the rest of the main carotenoids compounds (Figure 2), starting from carotenes and followed with xanthophylls, when α-carotene is converted into lutein while β-carotene is converted into cryptoxanthin, zeaxanthin, antheraxanthin, capsanthin, violaxanthin, and neoxanthin among other minor compounds.

As previously described, fruit and vegetables are the main sources of phytochemicals as carotenoids (Figure 2). According to Dias et al.,^70^ the worldwide consumed products richest in carotenoids are tomatoes, carrots, spinach, mandarins, maize, pumpkins, rosehips, and watermelons. Moreover, green leafy vegetables as kale, spinach, collards, and mustard greens are the richest vegetables in lutein.

From the individual point of view of each carotenoid compound, watermelons (*Citrullus lanatus*), rosehips (*Rosa canina*), tomatoes (*Solanum lycopersicum*), and especially the derivatives (processed foods obtained from tomato) are the richest foods in lycopene, characterized by their red color.^64^ Biosynthesized from this compound and characterized by their orange color, carrots (*Daucus carota*), peppers (*Capsicum annuum*), and pumpkins (*Cucurbita maxima*) are the richest compounds in α- and β-carotene.

On the one hand, lutein, characterized by its green color, is synthesized from α- carotene. This compound is abundant especially in green leafy vegetables, such as spinach (*Spinacia oleracea*) and Brassicaceae.^71,72^ On the other hand, cryptoxanthin and zeaxanthin are synthesized from β-carotene, and the richest foods in these compounds are pepper (*Capsicum annuum*), apricot (*Prunus armeniaca*), mandarin (*Citrus reticulata*), and tamarillo (*Solanum betaceum*) for the first one, while pepper (*Capsicum annuum*), maize (*Zea mays*), goji berries (*Lycium barbarum*), crabs (*Brachyura*), and salmon (*Salmo salar*) are the main sources of the second one.^73^ Also, algae and seaweed are important sources of these compounds as well as asteraxanthin and neoxanthin.^74^ In fact, although these foods have been widely used in oriental foods, and in the last several years, the incorporation of algae in new cuisine has introduced these “exotic” foods into our normal diet, making them more common.

As observed by the richness of these nutraceuticals in fruits and vegetables, Mediterranean and vegetarian diets could be the most abundant dietary patterns, which includes these phytochemical compounds.^75−77^ Furthermore, because of their solubility, carotenoids are more bioavailable when they are embedded in foods with a lipid phase (which generally are simultaneously consumed), like some fishes or yolks of eggs.

In this sense, the incorporation of oil during cooking helps to break down the cell walls of the plant making these compounds more bioavailable. In fact, in the case of lycopene, the heating during processing transform the double bond of the molecule from trans- to cis-, making it highly bioavailable in the human body.^78^

In this sense, nonedible parts, especially peels and leaves, which are the most important photoreceptors of the plant tissues, are one of the main sources of carotenoids, which also increase with high exposure to solar light and environmental changes. However, these parts of the plant are generally discarded to be used as extracts for livestock feed or as fertilizer in the fields.

Each year, billions of tones of fruits and vegetables are directly wasted. In fact, between 30 and 40% of the food supply chain is discarded in farms, supermarkets, and of course, in our own homes.^79^ This, without considering nonedible parts of fruits and vegetables that are discarded during food processing, as it is the case of the industry of fresh-cut and pre-cooked convenience food products, or the juice industry, among others.

For that reason, in last 20 years, new extraction methods have been developed to obtain carotenoid extracts, among other nutraceuticals, from fruit and vegetable waste for their subsequent incorporation into food products or supplements with important functional properties.

### Novel Approaches for the Extraction of Carotenoids from Food Waste

In this case, peels, seeds, roots, and leaves discarded by the food industry are the main sources for the extraction of carotenoids. As a matter of fact, the waste generated from them has got high water, oxygen, and nitrogen contents, which can lead to an important focus of contamination. Hence, as these food wastes are also rich in nutrients, they can be recovered and reused.^80^

Usually, the methods used for the extraction of plant carotenoids incorporate solvents, fermentations, enzymatic extractions, and new technologies such as microwave, ultrasound, cold plasma, or supercritical fluids. After extraction, to isolate pure compounds, these carotenoid extracts are purified by solid phase extraction and then separated by chromatographic techniques.^81^ In Table 3, modern methods used for the recovery of carotenoids are listed.

**Table 3:** Extraction of Carotenoids with Emerging Technologies

source of carotenoids	technology	extraction conditions	extraction yield	refs
tomato peels	supercritical fluid	extraction at 50–80 °C, pressures of 300–500 bar, and flow rates of 4–6 g, CO_2_ min^–1^ for 105 min	∼1200 mg lycopene kg^–1^ dry weight	(93)
			∼28 mg β-carotene kg^–1^ dry weight	
waste from peels of: tomato, tangerine, and orange	supercritical fluid		∼172 (tomato), ∼ 14.5 (tangerine), and ∼9.5 (orange) lycopene kg^–1^ dry weight	(91)
			∼10.6 (tomato), ∼ 29 (tangerine), and ∼50.4 (orange) mg β-carotene kg^–1^ dry weight	
waste from flesh and peels of: sweet potato, tomato, apricot, pumpkin, peach, apricot, and peppers	supercritical fluid	extraction at 59 °C, pressures of 350 bar, and flow rates of 15 g, CO_2_ min^–1^ for 30 min	high recovery percent (>90% for total carotenoids, β-carotene, and lycopene)	(92)
*Dunaliella salina*	supercritical fluid	extraction at 40–60 °C, pressures of 100–500 bar, and flow rates of 3 L CO_2_ min^–1^ for 90 min	∼115.4 mg carotenoids kg^–1^ dry weight	(94)
tomato peels	pulsed electric field	PEF pretreatment (5 kV cm^–1^, 5 kJ kg^–1^) with acetone or ethyl lactate (1:40 g mL^–1^) at 25 °C extraction time for 240 min	∼11820 mg lycopene kg^–1^ dry weight in acetone and ∼6311 mg lycopene kg^–1^ dry weight in ethyl lactate	(90)
tomato waste	pulsed electric field	PEF treatment (5 kV cm^–1^, 7 kJ kg^–1^) with hexane/ethanol (50:50) at 20 °C for 300 min	∼44 mg carotenoids kg^–1^ fresh weight	(89)
tomato pomace	ultrasound	ethyl lactate/ethyl acetate (7:3, 100 mL g^–1^) for 20 min	∼1335 mg lycopene kg^–1^ dry weight	(88)
pomegranate wastes	ultrasound	sunflower and soy oils (4 g mL^–1^) for 30 min	∼3.25 mg carotenoids kg^–1^ dry weight	(87)
dry tomato waste	ultrasound	sunflower, corn, and rapeseed oils (50 mg mL^–1^) 35 kHz at 20 °C for 50 min	∼34.8 (extra virgin sunflower oil), ∼ 38.4 (unrefined corn oil), and ∼35.4 (refined rapeseed oil) mg carotenoids kg^–1^ dry weight	(85)
tomato waste	microwave	300 W, 60 s, 95% ethanol, temperature <77 °C	∼57.4 mg lycopene kg^–1^ dry weight	(86)
			∼48.3 mg β-carotene kg^–1^ dry weight	
dry tomato waste	microwave	sunflower, corn, and rapeseed oils (50 mg mL^–1^) 700 W for 5 min	∼32.2 (extra virgin sunflower oil), ∼ 35.2 (unrefined corn oil), and ∼32.3 (refined rapeseed oil) mg carotenoids kg^–1^ dry weight	(85)
dry tomato waste	soaking for 7 days	maceration in sunflower, corn, and rapeseed oils (50 mg mL^–1^) at 20 °C for 7 days	∼40.2 (extra virgin sunflower oil), ∼ 40.9 (unrefined corn oil), and ∼37.6 (refined rapeseed oil) mg carotenoids kg^–1^ dry weight	(85)
tomato peel	thermal extraction	at 75 °C for 2 h combined to 50 mg of TiO_2_ nanoparticles per 250 g of tomato peel	∼7230 mg all-trans lycopene and ∼1570 mg cys-lycopene kg^–1^	(82)
crustacean wastes from blue crab and shrimp	solvents and oil extraction	ratio solvent/waste (2:1) at room temperature for 2 min	acetone: ∼6.6 (blue crab wastes) and ∼61.3 (shrimp wastes) mg carotenoids kg^–1^	(84)
		ratio oil/waste (2:1) at 70 °C for 2 h	sunflower oil: ∼ 0.21 (blue crab wastes) and ∼4 (shrimp wastes) mg carotenoids kg^–1^	
tomato pomace	water-induced hydrocolloidal complexation	separation: 7500 rpm for 20 min	∼108.1 mg carotenoids kg^–1^ fresh weight with a high purity level (>92%)	(83)
		recovery: 10000 rpm for 5 min		

For instance, in recent years, green technologies, such as the application of pulsed electric fields, ultrasound, and microwaves, have been highly developed to extract carotenoid compounds. However, conventional procedures, such as thermal extractions or application of polar solvents and oils are still commonly used to isolate carotenoids from the main food sources.

Martínez-Hernández, Castillejo, and Artés-Hernández^82^ have recently shown how using a 2 h thermal extraction at 75 °C and in combination with TiO_2_ it is possible to extract more than 4000 mg kg^–1^ from tomato peels, which is more than the 50% of the total lycopene content in this food byproduct. In addition, the application of the lycopene microspheres of this revalorized product has potentially increased the content in bioactive compounds in fresh-cut apples while maintaining their physico-chemical properties.

Also in tomato, Nagarajan et al.^83^ showed the high yield and versatility of the carotenoid-pectin complexation extraction method with a low economic cost and impact to the environment. In this sense, authors reached a 92% purity level after the water-induced hydrocolloidal complexation developed.

Similarly, using crustacean wastes from blue crab (*Portunus pelagicus*) and shrimp (*Penaeus semisulcatus*), Hooshmand et al.^84^ have optimized the extraction method of carotenoid compounds by using several polar solvents and oils. The obtained results showed that the use of acetone as an extraction solvent can report a yield of almost 7 mg of carotenoids kg^–1^ blue crab wastes and 61 mg of carotenoids kg^–1^ shrimp waste.

Because of its molecular hydrophobic characteristics, lycopene is easily extractable by using vegetable oils. As a matter of fact, Nour et al.^85^ have shown the oxidative stability of extracts from dry tomato waste by using several methods: microwave, ultrasound, and 7 days of maceration. The obtained results in this research showed that extra virgin sunflower, unrefined corn, and refined rapeseed oils are good extractors of carotenoids by the three technologies. Moreover, although no significant differences were appreciated among the chosen methods, soaked tomato waste reported a higher concentration of carotenoids in all the studied vegetable oils. Nevertheless, since the employed time in the maceration is too long for its application in the food industry, the use of microwaves and ultrasound have also shown mean extraction values of 35 mg of carotenoids per kg of dry tomato waste, approximately.

Similarly, according to Lasunon et al.,^86^ a 300 W microwave force is able to extract 57 mg of lycopene and 48 mg of β-carotene per kg of tomato waste. In fact, authors also demonstrated the high potential antioxidant content obtained from tomato waste powder.

From the other point of view, ultrasonication is also a green technology which has reported high carotenoid extraction yields. For instance, using vegetable oils, Goula et al.^87^ have developed comparative studies between ultrasound-assisted and a conventional solvent extraction with 60:40 hexane/isopropanol in pomegranate wastes. The obtained results have shown the optimum extraction yield using ultrasound technology was 3.3 mg of carotenoids per kg of dry pomegranate peels, which represented 94% of the total carotenoid content in the discarded material.

Moreover, improvements of the extraction yield have been shown by Ajlouni, Premier, and Tow^88^ in tomato pomace waste. In fact, 1335 mg of lycopene per kg of tomato pomace powder were obtained after extraction with ethyl lactate/ethyl acetate for 20 min of sonication. Lycopene extracts showed important scavenging capacities due to the high concentration of antioxidant compounds.

Also from tomato waste, pulsed electric fields have shown high extraction efficiencies for intracellular compounds, which has been assessed with water-soluble molecules and depends on the solvents employed and the procedure followed. As a matter of fact, Luengo, Álvarez, and Raso^89^ have extracted 44 mg of carotenoids per kg of fresh tomato waste mixed with hexane/ethanol, while Pataro et al.^90^ extracted 11 820 mg of lycopene per kg of dry tomato waste with acetone. Also, by mixing with ethyl lactate and using the same technology (pulsed electric fields), the lycopene extraction yield was reduced almost to half (6 311 mg of lycopene per kg of dry tomato waste).

Furthermore, in the past years, the application of supercritical fluids technology has demonstrated high extraction yields in food wastes rich in carotenoids. Rubashvili et al.^91^ has demonstrated high efficiency rates by using supercritical fluids on food byproducts from tomatoes, tangerines, and oranges. As a matter of fact, Andrade Lima et al.^92^ have developed a standard method to reach carotenoid recovery percentages higher than 90%, especially for β-carotene and lycopene, in different food matrixes: waste from flesh and peels from sweet potatoes, tomatoes, apricots, pumpkins, peaches, and bell peppers.

Specifically, to extract carotenoids in tomato peels, supercritical fluids have also been demonstrated to extract 1200 mg of lycopene and 28 mg of β-carotene kg^–1^ of dry product. Interestingly, accordingly in Kehili et al.,^93^ supercritical CO_2_ exhibited an important quenching activity. For that, the Tunisian industrial tomato byproduct can have promising applications in the food industry.

In addition, this extraction procedure has also shown important yields in *Dunaliella salina* microalgae. Hence, suggested by Pour Hosseini et al.,^94^ the highest carotenoid extraction yields were obtained at 400 bar and 55 °C, with mean values of 115 mg of carotenoids per kg of dry algae.

Along with the technologies aforementioned, some authors have investigated the potential of solid-state fermentation to enrich food byproducts with bioactive compounds in order to promote a greater utilization of these byproducts in the pharmaceutical and food industries. In this regard, Dulf et al.^95^ observed that the amount of β-carotene in grape pomace increased after 12 days of solid-state fermentation using two filamentous fungal strains (*Actinomucor elegans* and *Umbelopsis isabelline*). In this sense, the incorporation of such purified compounds throughout green technologies can also show potential health benefits in the human body.

### Functional and Bioactive Properties of Carotenoids

The main known function of carotenoids in humans is to serve as precursors to vitamin A (retinol) and in gene regulation linked to many physiological and developmental processes (retinoic acid).^96^ β-carotene (BC) is the main provitamin A carotenoid in the human diet. Other carotenoids are β-cryptoxanthin and α-carotene. Nonprovitamin carotenoids A (including zeaxanthin, lycopene, and lutein are abundant in the human body) and provitamin A can act as antioxidants and photoprotective filters for blue light.^97^ Currently, there is a new perspective on the role of carotenoids and their derivative products that connects these compounds with control of the accumulation of body fat and adipocyte biology, with possible implications for the treatment of obesity.^98,99^

In addition, they exert antioxidant effects, but individual carotenoids may also act as a pro-vitamin A function (β-carotene) or constitute a macular pigment in the eye (lutein/zeaxanthin). Therefore, an intake recommendation of lutein rich foods should be made for the general population. Carotenoids also produce improvements in some types of cancer prevention, cardiovascular health, and cognitive function. The main benefits of carotenoids can be explained by their antioxidant capacity,^100^ although, they may also act through others mechanisms.^101,102^ For example, carotenoids may help prevent heart disease by decreasing the oxidation of low-density lipoproteins or avoiding their formation,^103,104^ by interrupting gap-junctional communication, or avoiding the abnormal growth of cancer cells and acting against certain types of cancer.^105^

Moreover, there is evidence that carotenoids may have effects on cognitive functioning, and this effect is related to antioxidant activity.^106^ In this sense, a long-term β-carotene supplementation with 50 mg on alternate days maintained cognitive performance in a healthy population.^107^

Regarding the effects of carotenoids on cancer, there is evidence that lycopene is related to prostate cancer, because it has been found in high concentrations within the prostate gland,^108^ reporting an inverse association between prostate cancer and lycopene intake.^109^ In this same line, a meta-analysis of 42 epidemiological studies found that dietary lycopene consumption was significantly and inversely correlated with prostate cancer.^110^ Plasma lycopene levels are reduced in patients with nonalcoholic fatty liver, a disorder which is associated with hepatocellular carcinoma. Other studies have reported the relationship between lycopene levels and hepatocellular carcinoma disease.^111^ This effect has been shown in animal studies in which lycopene supplementation for 24 weeks had a protection against hepatic tumorigenesis.^112^

In addition, carotenoids may have beneficial effects on cardiovascular disease because they have an effect on oxidative stress, inflammation, dyslipidaemia, and thrombosis. Astaxanthin has been reported to improve blood lipid profiles and to reduce low-density lipoprotein peroxidation. Several studies have shown that astaxanthin improves lipid profiles^104^ because it reduces oxidation of low-density lipoprotein and its contribution to develop.^103^ Another carotenoid, lycopene, shows potent antioxidant activity and reduces serum cholesterol in animal studies.^113,114^

Epidemiological studies have associated circulating high levels of β-carotene and other carotenoids with a lower risk of suffering from metabolic and cardiovascular diseases,^115^ and this is mainly due to a high consumption of fruits and vegetables. Taking into account that the fabric adipose is an important deposit of carotenoids and retinol and that body fat is a determining factor in susceptibility to many metabolic disorders,^116^ it is conceivable that the potential beneficial effects of carotenoids and retinoids on health are closely linked to the modulation of associated phenomena to “adiposopathy” fat. In fact, numerous preclinical studies indicate that carotenoids and derived apocarotenoids (retinoids and others) modulate key aspects of adipose tissue biology, including differentiation, hypertrophic expansion, capacity for fat oxidation and thermogenesis, and secretory function of the adipocytes. Specifically, in vitro^117^ and animal studies have shown that carotenoids (apo-carotenoids and retinoids) have a beneficial effect on adipocyte differentiation.^118,119^ Moreover, the antioxidant potential of carotenoids might also be related to its effects on obesity and weight management.^118^ In addition, carotenoids have an effect on infant nutrition.^120^ Zeaxanthin and lutein have been found in regions of the infant brain that are specialized for language, visual processing, learning, and memory.^120^

## Chlorophylls

### Sources of Chlorophylls

Chlorophylls are oil-soluble, amphiphilic pigments with a green color, which are extensively distributed in several plants, algae, and cyanobacteria.^121^ Chlorophylls can be found in plants with two different structures: Chlorophyll-a, which has a methyl (−CH_3_) group at the 7-carbon position, and chlorophyll-b, which has an aldehyde (−CHO) group at the 7-carbon position.^4^ Due to this structural difference, chlorophylls have different colors. Thus, chlorophyll-a has a blue-green color, while chlorophyll-b shows a blue-yellow color.^122^ Both chlorophyll-a and chlorophyll-b coexist in plants in a ratio of 3:1.^123^

The main sources of chlorophyll are the leafy vegetables such as spinach (*Spinacia oleracea*), lettuce (*Lactuca sativa*), and broccoli (*Brassica oleracea*) among others. For spinach, Derrien et al.^124^ carried out a study to determine the chlorophyll content of spinach byproducts. These authors reported that the chlorophyll content (chlorophyll-a + chlorophyll-b) in the samples was 112.8 mg/100 g fresh weight. In a similar study, Zhang et al.^125^ investigated the chlorophyll content of spinach that did not have the commercial criteria and therefore were destined for waste. They reported that the chlorophyll-a content was between 10.93 and 14.62 μg/mL of extract, while the chlorophyll-b content ranged between 4.79 and 6.13 μg/mL of extract. In the same way, Chemat et al.^126^ informed that the chlorophyll content of spinach leaves was 800 μg/mL of extract. Another vegetal that generates large amounts of waste and can be a source of chlorophylls is lettuce. Thus, in an interesting study, Agüero et al.^127^ analyzed the total chlorophyll content of external leaves, which normally are discarded, of butter lettuce (*L. sativa* var. Lores) cultivated in Argentina. These authors reported that the chlorophyll content was of 35.65 mg/100 g of fresh weight. More recently, Kowalczyk et al.^128^ analyzed the chlorophyll content of lettuce of the butterhead type cv. Omega’ F1. They informed that the chlorophyll content found in the lettuce leaves was 24.0 mg/100 g fresh weigh, which corresponds to 18.1 mg/100 g fresh weight of chlorophyll-a and 5.9 mg/100 g fresh weight of chlorophyll-b.

Broccoli also generated a great amount of waste. Thus, only around 10–15% of the total aerial biomass of the plant, which represents 47% of the product, is consumed. Another source of waste is the floret (which represents 15% of the product) that did not have the commercial criteria. Thus, both leaves and floret broccoli wastes are recognized as a great source of chlorophylls. In this sense, Liu et al.^129^ reported that the chlorophyll-a content of broccoli (*cv.* green magic) leaf was 447.79 mg/100 g dry weight, while the chlorophyll-b content was 78.09 mg/100 g dry weight, values higher than found in the stems and florets. More recently, Ferreira et al.^130^ carried out a work to analyzed the chlorophyll content in broccoli byproducts composed of stalks and leaves from fresh broccoli submitted for freeze-drying. These authors informed that the chlorophyll-a content was 349.23 mg/100 g dry weight, while the chlorophyll-b content was 53.05 mg/100 g dry weight. Similarly, Borja-Martinez et al.^131^ analyzed the chlorophyll content of broccoli byproducts (leaves and stems) from the *cv.* Parthenon and Naxos. These authors informed that in *cv.* parthenon byproducts, the total chlorophyll was 32.64 mg/g dry weight, while in cv. naxos the total chlorophyll was 40.89 mg/g dry weight.

Several others vegetable wastes can be considered as sources of chlorophylls. In this way, Zeyada et al.^132^ analyzed the total chlorophylls content in several agro-industrial byproducts including watermelon (*Citrullus lanatus*) and cucumber (*Cucumis sativus*) peels. The values reported were for watermelon 528 mg/100 g dry weight and for cucumber 346 mg/100 g dry weight. Singh et al.^133^ determined the total chlorophyll content in the byproducts of kale (*Brassica oleracea* L.) of three commercial cultivars, such as Siberian Kale, Khanyari, and Japanese Green, which had values of 134.92, 157.01, and 169.89 mg/100 g dry weight, respectively. Ruiz-Cano et al.^134^ investigated the chlorophylls content in artichoke (*Cynara scolymus*, L. cv. blanca de tudela) byproducts, which consist of the raw outer bracts and stems removed mechanically from the artichoke heads. These authors reported that the total chlorophyll content of artichoke byproducts was 27.50 mg/100 g dry weight. Fundo et al. (2017)^135^ analyzed the total chlorophyll content of cantaloupe melon (*Cucumis melon* L. var. reticulatus) waste composed of peels and seeds. They reported that the total chlorophyll content in the melon peel was 7.89 mg/100 g distributed by chlorophylls-a (4.58 mg/100 g) and chlorophylls-b (3.29 mg/100 g). More recently, Carbone et al.^136^ examined the total chlorophylls content present in kiwi (*Actinidia deliciosa*, cv. “Hayward”) juice pomace and reported a concentration of 5.90 mg/100 g fresh weight. Chaiareekitwat et al. (2022)^137^ reported that in cassava leaves (*Manihot esculenta* Crantz), the total chlorophyll (sum of chlorophyll-a and chlorophyll-b) content ranged from 326.27 to 747.86 mg/100 g dry weight depending on the plant age, cultivar analyzed, and position.

Another very important source of chlorophylls are the microalgae, mainly those belonging to genera *Arthrospira* and *Chlorella*. For genus *Arthrospira*, Kent et al.^138^ investigated the total chlorophylls content of commercial *Arthrospira platensis* strains from China. These authors reported values of total chlorophylls of 1233 mg/100 g dry weight. Similarly, Aouir et al.^139^ analyzed the chlorophylls content of *A. platensis* strains obtained from the Pacific Ocean. These authors reported a content of total chlorophylls of 849 mg/100 g dry weight corresponding to 639 mg/100 g of chlorophyll-a and 210 mg/100 g of chlorophyll-b. More recently, Tavanandi and Raghavarao^140^ carried out a research study to analyze the chlorophylls contents from spent biomass, left after recovery of phycobiliproteins of *A. platensis*. They informed that the total chlorophyll content was 595 mg/100 g dry weight. In the case of genus *Chlorella*, Kong et al.^141^ reported that the total chlorophyll content of *Chlorella vulgaris* residue after lipid extraction is 3520 mg/100 g. Guo and Fang^142^ proposed a study to analyze the chlorophyll content present in *Chlorella pyrenoidosa* subjected to different light types. They found that the values were comprised between 1480 and 1980 mg/100 g dry weight.

In view of these studies, it can be said that, in general, the residues from plants are a good source of chlorophylls; however, it is the residues from microalgae where the greatest concentration of these pigments can be found.

### Novel Approaches for the Extraction of Chlorophylls from Food Waste

Generally, to extract chlorophylls from vegetables or microalgae matrixes, different conventional methodologies such as maceration and Soxhlet extraction techniques with organic or inorganic solvents have been widely used.^122^ Nevertheless, it is important to note that the conventional extraction methodologies show several deficiencies including low extraction yield, long extraction times, very complex processes, and elevated capital investments.^4^ To avoid these deficiencies, several novel technologies are being used which could be classified as green extraction methodologies that include supercritical fluid extraction, ultrasound assisted extraction, microwave assisted extraction, pulsed-electric field-assisted extraction, and enzyme-assisted extraction.^81,143^

In regards to the supercritical fluid extraction methodology, in a research study with the byproducts obtained from two different broccoli cultivars, Borja-Martinez et al.^131^ reported that the chlorophylls content increased 8.72 and 32.64% in cultivars parthenon and naxos, respectively, when using supercritical fluid extraction with ethanol at a pressure of 400 bar in comparison with conventional extraction methodology. Similarly, Derrien et al.^144^ informed that the chlorophylls extraction obtained from spinach byproducts increased 50% with respect to conventional methodology when using the supercritical CO_2_ extraction methodology with the parameters of temperature of 56 °C, extraction time of 3.6 h, pressure of 39 MPa, and 10% ethanol as cosolvent. In a previous study, Derrien et al.^124^ reported that using supercritical CO_2_ extraction with 93% ethanol concentration for 4.3 h at 43 °C and a solvent to raw material ratio of 1/66, the chlorophylls extraction from spinach byproducts increased 96% when compared with conventional extraction using acetone.

In regards to ultrasound-assisted extraction methodology, Chemat et al.^126^ mentioned that the extraction of chlorophylls from spinach byproducts is 4-fold higher when using ultrasound than when using a traditional maceration. Previously, Kong et al.^141^ conducted an investigation of the extraction of chlorophyll from *Chlorella vulgaris* residue after lipid separation using ultrasound-assisted extraction. These authors found that using an extraction temperature of 61.4 °C, an extraction time of 78.7 min, with an ethanol volume of 79.4%, and a fixed ultrasonic power of 200 W, the extraction rate reached up to 88.9%. These results confirm those exposed by Kwang et al.^145^ who mentioned that ultrasound-assisted extraction resulted in an increased yield of chlorophyll content present from *Chlorella vulgaris* (8.83 mg/g) when compared to conventional extraction methods like maceration (6.84 mg/g).

Another very interesting novel methodology to extract bioactive compounds in general and chlorophylls in particular is microwave assisted extraction. Thus, Guo et al.^146^ studied the total chlorophyll content of sugar cane wastes using microwave assisted extraction and ethanol as the solvent. When compared with the traditional extraction methodologies, microwave assisted extraction was more effective with 27.7 mg/100 g of total chlorophylls in 60 min, while in conventional method 25.9 mg/100 g total chlorophylls in 240 min was obtained. Michalak et al.^147^ studied the chlorophylls content of algae biomass (*Polysiphonia*, *Ulva*, *Cladophora*) from the Baltic Sea extracted by means of microwave-assisted extraction. They reported that with these extraction conditions 1000 W, liquid/solid ratio (3/1) extraction time 30 min, and 60 °C of extraction temperature, the cholorphylls content extraction increased to 12.5%. Nguyen et al.^148^ assessed the effect of microwave-assisted extraction conditions on the chlorophyll content of pandan (*Pandanus amaryllifolius* Roxb.) leaf. These authors reported that these conditions: acetone as solvent at 90 °C, pandan powder/acetone ratio of 1:30, microwave capacity of 300 W, time 2 min were the best parameters for the highest extraction efficiency (chlorophyll-a, chlorophyll-b, and total chlorophyll content were, respectively, 9.4278 μg/mL, 4.2460 μg/mL, and 13.6738 μg/mL).

Pulsed electric field-assisted extraction is a very interesting methodology to obtain bioactive compounds, like chlorophylls from vegetable or algae tissues. In this sense, Luengo et al.^149^ analyzed the effect of pulsed electric field treatments on the extraction of chlorophylls of the microalgae *Chlorella vulgaris*. They found that after pulsed electric field treatments at 20 kV/cm for 75 μs, the extraction yield for chlorophyll-a and chlorophyll-b increased 1.6, and 2.1 times, respectively. Zhang et al.^125^ investigated the effect of pulse-electric field-assistance on the chlorophylls content present in spinach byproducts. These authors informed that at 20 °C, 60 min, and an electric field strength of 26.7 kV/cm, the chlorophylls extraction increased to 24.63%. Pataro et al.^150^ evaluated the effect of pulsed electric fields treatment on the extractability of chlorophyll-a from microalgae *Nannochloropsis oceanica*. These authors informed that after a pulsed electric field treatment at 10 kV/cm and 100 kJ/kg, the extraction yield for chlorophyll-a increased by 1.4 times.

Enzyme-based extraction of pigments from vegetables and algae is a potential alternative to conventional extraction methods. Therefore, Özkan and Bilek^151^ analyzed the effect of enzyme concentration treatment, temperature, and time on total chlorophyll content (TCC) present in spinach. These authors concluded that enzyme-assisted extraction of zinc-chlorophyll derivatives from spinach pulp under optimized conditions (8% enzyme concentration, 45 °C, and 30 min) had a significant increase in the yield by 39%.

### Functional and Bioactive Properties of Chlorophylls

As mentioned above, chlorophyll is a very important bioactive compound present in vegetables and algae. This biomolecule has several properties as color pigment as well as its physiological role in plants.^152^ Additionally, chlorophyll has been related with several health benefits as a nutraceutical agent with antioxidant, antimutagenic, and anti-inflammatory.^153^

The antioxidant activity of chlorophylls and chlorophyll derivatives has been the subject of several in vitro studies.^154−157^ Thus, Lanfer-Marquez et al.^154^ reported that chlorophyll-a obtained from spinach leaves showed lower antioxidant capacity measured with the β-carotene bleaching method than that the chlorophylls derivatives including pheophorbide-a, pheophytin-b, pheophorbide-b, and Cu-chlorophyllin with values of inhibition ranging between 42% and 85% at concentrations of 200 mg of BHT equivalents/kg. In a similar work, Hsu et al.^157^ studied the antioxidant activity of chlorophyll-a and chlorophyll-b extracted from spinach leaves as well as the chlorophyll derivatives using the DPPH radical scavenging assay. They found that chlorophyll derivatives had higher antioxidant activity than chlorophylls. Thus, at concentrations of 200 μM chlorophyll derivatives showed scavenging effects around of 50% while chlorophylls were around 40%. Similarly, Kang et al.^156^ analyzed the antioxidant activity of chlorophyll and chlorophyll derivatives including pheophytins and zinc-pheophytins extracted from spinach leaves using a DPPH radical scavenging assay and β-carotene bleaching test. They reported that in the DPPH assay, zinc-pheophytins had the higher antioxidant capacity (22.09%) followed by chlorophylls (13.89%) and pheophytins (12.79%), nevertheless all values were lower than Trolox (29.49%) which was used as a positive control. For inhibition of the β-carotene bleaching test, zinc-pheophytins showed the higher antioxidant activity (66.43%) followed by chlorophylls (49.63%), BHT (31.75%) used as positive control, and finally pheophytins (13.44%).

Numerous works have been conducted to assess the effectiveness of chlorophylls and chlorophylls derivatives as an antiproliferative, anti-invasive, and pro-apoptotic agent in several cancer cell lines and animal models.^158,159^ Therefore, Cheng and Lee^158^ informed that three pheophorbide compounds, which are produced by the breakdown of chlorophyll isolated from the leaves and stems of *Clerodendrum calamitosum*, demonstrated strong cytotoxicity against human lung carcinoma (A549), ileocecal carcinoma (HCT-8), kidney carcinoma (CAKI-1), breast adenocarcinoma (MCF-7), malignant melanoma (SK-MEL-2), ovarian carcinoma (1A9), and epidermoid carcinoma of the nasopharynx (KB). de Vogel et al. (2005)^159^ reported that rats which were fed with spinach showed a chlorophyll concentration of 1.2 mmol/kg reduced the formation of cytotoxic heme metabolites and decreased the colon cancer risk.

In reference to anti-inflammatory properties, Kang et al.^156^ reported that chlorophyll and chlorophyll derivatives including pheophytins and zinc-pheophytins extracted from spinach significantly suppressed the lipopolysaccharide-induced nitric oxide production in RAW 264.7 cells in a dose-dependent manner. The tested Zn-pheophytin concentrations did not show cell toxicity.

## Conclusions

Globally, there has been a significant rise in the amount of food waste generated because of increased industrial food production to meet the consumer demand. These food wastes, particularly fruit and vegetable byproducts, are potential sources of natural pigments including carotenoids, anthocyanins, betalains, and chlorophylls. Incorporation of natural pigments in food products would improve the appearance of food products in addition to providing potential positive effects on human health. Therefore, recovery of natural pigments from food wastes is important for both economical and environmental reasons. Conventional methods that are used to extract natural pigments from food wastes are time-consuming, expensive, and unsustainable. In addition, natural pigments are sensitive to high temperatures and prolonged processing times that are applied during conventional treatments. To overcome these limitations, several novel food processing approaches have been applied to recover natural pigments from food wastes. Pulsed electric field, ultrasound-, microwave-, and high-pressure-assisted-extraction methods are some important examples of these novel food processing methods. Extraction of natural pigments using novel food processing technologies have several advantages including better isolation, higher selectivity, reduced energy consumption, and low environmental impact. However, these methods also have some limitations including high equipment cost, low possibility of scale-up, and industrial applications. The polarity of the natural pigment and sensitivity toward thermal treatment also plays a critical role. In this respect, pulsed electric field treatment could be suitable for the extraction of less polar natural pigments, while high-pressure-assisted extraction could be useful for heat sensitive pigments. In most cases, ultrasound-assisted extraction is considered a suitable pretreatment.

Overall, there is a need for replacement of conventional extraction methods with novel approaches. However, as mentioned above, investment on new equipment is a challenge and each emerging innovation requires considerable examination. This would lead to high-cost investments and industrial risks. On the other hand, depending on the context, these challenges may turn out to be advantageous as nowadays consumers demand products with high sensorial quality, which could be achieved using the novel food processing approaches. Another challenge for future research involves the clarification of legislative status. This issue may be hard to handle as the manufacturer needs to ensure that the product produced with the novel food processing method conforms to the conventional processes. Furthermore, for the commercialization of these products, there is a need for approval and certification regarding the fact that the pigments still maintain the desired bioactive properties despite the fact that they are recovered from the food waste.

## References

[ref1] King T.; Cole M.; Farber J. M.; Eisenbrand G.; Zabaras D.; Fox E. M.; Hill J. P. Food Safety for Food Security: Relationship between Global Megatrends and Developments in Food Safety. Trends Food Sci. Technol. 2017, 68, 160–175. 10.1016/j.tifs.2017.08.014.

[ref2] Kumari B.; Tiwari B. K.; Hossain M. B.; Brunton N. P.; Rai D. K. Recent Advances on Application of Ultrasound and Pulsed Electric Field Technologies in the Extraction of Bioactives from Agro-Industrial By-Products. Food Bioprocess Technol. 2018, 11 (2), 223–241. 10.1007/s11947-017-1961-9.

[ref3] Galanakis C. M. Recovery of High Added-Value Components from Food Wastes: Conventional, Emerging Technologies and Commercialized Applications. Trends Food Sci. Technol. 2012, 26 (2), 68–87. 10.1016/j.tifs.2012.03.003.

[ref4] Sharma M.; Usmani Z.; Gupta V. K.; Bhat R. Valorization of Fruits and Vegetable Wastes and By-Products to Produce Natural Pigments. Crit. Rev. Biotechnol. 2021, 41 (4), 535–563. 10.1080/07388551.2021.1873240. 33634717

[ref5] Putnik P.; Lorenzo J.; Barba F.; Roohinejad S.; Rezek Jambrak A.; Granato D.; Montesano D.; Bursac Kovacevic D. Novel Food Processing and Extraction Technologies of High-Added Value Compounds from Plant Materials. Foods 2018, 7 (7), 106 10.3390/foods7070106. PMC6069231 29976906

[ref6] Nieto G. Anthocyanins: Health Benefits and Potential Use as Functional Ingredient: A Review. In Anthocyanins. Antioxidant Properties, Sources and Health Benefits; Rodriguez J. M. L., Barba F. J., Munekata P. E. S., Eds.; Nova Science Publishers: New York, 2020; pp 1–27.

[ref7] Kamiloglu S.; Capanoglu E.; Grootaert C.; Van Camp J. Anthocyanin Absorption and Metabolism by Human Intestinal Caco-2 Cells-a Review. Int. J. Mol. Sci. 2015, 16 (9), 21555–21574. 10.3390/ijms160921555. 26370977 PMC4613267

[ref8] Struck S.; Plaza M.; Turner C.; Rohm H. Berry Pomace - A Review of Processing and Chemical Analysis of Its Polyphenols. Int. J. Food Sci. Technol. 2016, 51 (6), 1305–1318. 10.1111/ijfs.13112.

[ref9] Costa E.; da Silva J. F.; Cosme F.; Jordão A. M. Adaptability of Some French Red Grape Varieties Cultivated at Two Different Portuguese Terroirs: Comparative Analysis with Two Portuguese Red Grape Varieties Using Physicochemical and Phenolic Parameters. Food Res. Int. 2015, 78, 302–312. 10.1016/j.foodres.2015.09.029. 28433296

[ref10] Bordiga M.; Travaglia F.; Locatelli M. Valorisation of Grape Pomace: An Approach That Is Increasingly Reaching Its Maturity - a Review. Int. J. Food Sci. Technol. 2019, 54 (4), 933–942. 10.1111/ijfs.14118.

[ref11] Somavat P.; Li Q.; de Mejia E. G.; Liu W.; Singh V. Coproduct Yield Comparisons of Purple, Blue and Yellow Dent Corn for Various Milling Processes. Ind. Crops Prod. 2016, 87, 266–272. 10.1016/j.indcrop.2016.04.062.

[ref12] Serrano-Díaz J.; Sánchez A. M.; Maggi L.; Martínez-Tomé M.; García-Diz L.; Murcia M. A.; Alonso G. L. Increasing the Applications of *Crocus Sativus* Flowers as Natural Antioxidants. J. Food Sci. 2012, 77 (11), C1162–C1168. 10.1111/j.1750-3841.2012.02926.x. 23057806

[ref13] Albuquerque B. R.; Pinela J.; Barros L.; Oliveira M. B. P. P.; Ferreira I. C. F. R. Anthocyanin-Rich Extract of Jabuticaba Epicarp as a Natural Colorant: Optimization of Heat- and Ultrasound-Assisted Extractions and Application in a Bakery Product. Food Chem. 2020, 316, 126364 10.1016/j.foodchem.2020.126364. 32058190

[ref14] Backes E.; Pereira C.; Barros L.; Prieto M. A.; Genena A. K.; Barreiro M. F.; Ferreira I. C. F. R. Recovery of Bioactive Anthocyanin Pigments from *Ficus Carica* L. Peel by Heat, Microwave, and Ultrasound Based Extraction Techniques. Food Res. Int. 2018, 113, 197–209. 10.1016/j.foodres.2018.07.016. 30195514

[ref15] Dranca F.; Oroian M. Total Monomeric Anthocyanin, Total Phenolic Content and Antioxidant Activity of Extracts from Eggplant (Solanum Melongena L.) Peel Using Ultrasonic Treatments. J. Food Process Eng. 2017, 40 (1), e12312 10.1111/jfpe.12312.

[ref16] Chen L.; Yang M.; Mou H.; Kong Q. Ultrasound-Assisted Extraction and Characterization of Anthocyanins from Purple Corn Bran. J. Food Process. Preserv. 2018, 42 (1), e13377 10.1111/jfpp.13377.

[ref17] Machado A. P. D. F.; Pereira A. L. D.; Barbero G. F.; Martínez J. Recovery of Anthocyanins from Residues of *Rubus Fruticosus Vaccinium Myrtillus* and *Eugenia Brasiliensis* by Ultrasound Assisted Extraction, Pressurized Liquid Extraction and Their Combination. Food Chem. 2017, 231, 1–10. 10.1016/j.foodchem.2017.03.060. 28449984

[ref18] Garcia-Mendoza M. d. P.; Espinosa-Pardo F. A.; Baseggio A. M.; Barbero G. F.; Marostica Junior M. R.; Rostagno M. A.; Martinez J. Extraction of Phenolic Compounds and Anthocyanins from Juç ara (*Euterpe Edulis* Mart.) Residues Using Pressurized Liquids and Supercritical Fluids. J. Supercrit. Fluids 2017, 119, 9–16. 10.1016/j.supflu.2016.08.014.

[ref19] Machado A. P. D. F.; Rueda M.; Barbero G. F.; Martín Á.; Cocero M. J.; Martínez J. Co-Precipitation of Anthocyanins of the Extract Obtained from Blackberry Residues by Pressurized Antisolvent Process. J. Supercrit. Fluids 2018, 137, 81–92. 10.1016/j.supflu.2018.03.013.

[ref20] Romero-Díez R.; Matos M.; Rodrigues L.; Bronze M. R.; Rodríguez-Rojo S.; Cocero M. J. J.; Matias A. A. A. Microwave and Ultrasound Pre-Treatments to Enhance Anthocyanins Extraction from Different Wine Lees. Food Chem. 2019, 272, 258–266. 10.1016/j.foodchem.2018.08.016. 30309541

[ref21] Brianceau S.; Turk M.; Vitrac X.; Vorobiev E. Combined Densification and Pulsed Electric Field Treatment for Selective Polyphenols Recovery from Fermented Grape Pomace. Innov. Food Sci. Emerg. Technol. 2015, 29, 2–8. 10.1016/j.ifset.2014.07.010.

[ref22] Zhou Y.; Zhao X.; Huang H. Effects of Pulsed Electric Fields on Anthocyanin Extraction Yield of Blueberry Processing By-Products. J. Food Process. Preserv. 2015, 39 (6), 1898–1904. 10.1111/jfpp.12427.

[ref23] Ahmadian-Kouchaksaraie Z.; Niazmand R. Supercritical Carbon Dioxide Extraction of Antioxidants from *Crocus Sativus* Petals of Saffron Industry Residues: Optimization Using Response Surface Methodology. J. Supercrit. Fluids 2017, 121, 19–31. 10.1016/j.supflu.2016.11.008.

[ref24] Machado A. P. D. F.; Pasquel-Reátegui J. L.; Barbero G. F.; Martínez J. Pressurized Liquid Extraction of Bioactive Compounds from Blackberry (Rubus Fruticosus L.) Residues: A Comparison with Conventional Methods. Food Res. Int. 2015, 77, 675–683. 10.1016/j.foodres.2014.12.042.

[ref25] Saldaña M. D. A.; Martinez E. R.; Sekhon J. K.; Vo H. The Effect of Different Pressurized Fluids on the Extraction of Anthocyanins and Total Phenolics from Cranberry Pomace. J. Supercrit. Fluids 2021, 175, 105279 10.1016/j.supflu.2021.105279.

[ref26] Kurtulbas Sahin E.; Bilgin M.; Sahin S. Recovery of Anthocyanins from Sour Cherry (*Prunus Cerasus* L.) Peels via Microwave Assisted Extraction: Monitoring the Storage Stability. Prep. Biochem. Biotechnol. 2021, 51 (7), 686–696. 10.1080/10826068.2020.1852418. 33275494

[ref27] Varadharajan V.; Shanmugam S.; Ramaswamy A. Model Generation and Process Optimization of Microwave-Assisted Aqueous Extraction of Anthocyanins from Grape Juice Waste. J. Food Process Eng. 2017, 40 (3), e12486 10.1111/jfpe.12486.

[ref28] Ferreira L. F.; Minuzzi N. M.; Rodrigues R. F.; Pauletto R.; Rodrigues E.; Emanuelli T.; Bochi V. C. Citric Acid Water-Based Solution for Blueberry Bagasse Anthocyanins Recovery: Optimization and Comparisons with Microwave-Assisted Extraction (MAE). Lwt 2020, 133, 110064 10.1016/j.lwt.2020.110064.

[ref29] Coelho M.; Silva S.; Costa E.; Pereira R. N.; Rodrigues A. S.; Teixeira J. A.; Pintado M. Anthocyanin Recovery from Grape By-Products by Combining Ohmic Heating with Food-Grade Solvents: Phenolic Composition, Antioxidant, and Antimicrobial Properties. Molecules 2021, 26 (13), 3838 10.3390/molecules26133838. 34202440 PMC8270259

[ref30] Pereira R. N.; Coelho M. I.; Genisheva Z.; Fernandes J. M.; Vicente A. A.; Pintado M. E.; Teixeira e. J. A. Using Ohmic Heating Effect on Grape Skins as a Pretreatment for Anthocyanins Extraction. Food Bioprod. Process. 2020, 124, 320–328. 10.1016/j.fbp.2020.09.009.

[ref31] Amaya-Chantaca D.; Flores-Gallegos A. C.; Iliná A.; Aguilar C. N.; Sepúlveda-Torre L.; Ascacio-Vadlés J. A.; Chávez-González M. L. Comparative Extraction Study of Grape Pomace Bioactive Compounds by Submerged and Solid-State Fermentation. J. Chem. Technol. Biotechnol. 2021, 10.1002/jctb.6977.

[ref32] Dulf F. V.; Vodnar D. C.; Dulf E. H.; Diaconeasa Z.; Socaciu C. Liberation and Recovery of Phenolic Antioxidants and Lipids in Chokeberry (Aronia Melanocarpa) Pomace by Solid-State Bioprocessing Using Aspergillus Niger and Rhizopus Oligosporus Strains. Lwt 2018, 87, 241–249. 10.1016/j.lwt.2017.08.084.

[ref33] Azeredo H. M. C. Betalains: Properties, Sources, Applications, and Stability - A Review. Int. J. Food Sci. Technol. 2009, 44 (12), 2365–2376. 10.1111/j.1365-2621.2007.01668.x.

[ref34] Leong H. Y.; Show P. L.; Lim M. H.; Ooi C. W.; Ling T. C. Natural Red Pigments from Plants and Their Health Benefits: A Review. Food Rev. Int. 2018, 34 (5), 463–482. 10.1080/87559129.2017.1326935.

[ref35] Carrillo C.; Wilches-Pérez D.; Hallmann E.; Kazimierczak R.; Rembiałkowska E. Organic versus Conventional Beetroot. Bioactive Compounds and Antioxidant Properties. LWT 2019, 116, 108552 10.1016/j.lwt.2019.108552.

[ref36] Carrillo C.; Rey R.; Hendrickx M.; del Mar Cavia M.; Alonso-Torre S. Antioxidant Capacity of Beetroot: Traditional vs Novel Approaches. Plant Foods Hum. Nutr. 2017, 72 (3), 266 10.1007/s11130-017-0617-2. 28620796

[ref37] Neelwarne B.; Halagur S. B. Red Beet: An Overview. In Red Beet Biotechnology: Food and Pharmaceutical Applications; Springer, 2012; pp 1–43, 10.1007/978-1-4614-3458-0_1.

[ref38] Ubeira-Iglesias M.; Wilches-Pérez D.; Cavia M. M.; Alonso-Torre S.; Carrillo C. High Hydrostatic Pressure Processing of Beetroot Juice: Effects on Nutritional, Sensory and Microbiological Quality. High Press. Res. 2019, 39 (4), 691 10.1080/08957959.2019.1666842.

[ref39] Guldiken B.; Toydemir G.; Nur Memis K.; Okur S.; Boyacioglu D.; Capanoglu E. Home-Processed Red Beetroot (Beta Vulgaris L.) Products: Changes in Antioxidant Properties and Bioaccessibility. Int. J. Mol. Sci. 2016, 17 (6), 858 10.3390/ijms17060858. PMC4926392 27258265

[ref40] Wootton-Beard P. C.; Ryan L. A Beetroot Juice Shot Is a Significant and Convenient Source of Bioaccessible Antioxidants. J. Funct. Foods 2011, 3 (4), 329–334. 10.1016/j.jff.2011.05.007.

[ref41] Ben-Othman S.; Jõudu I.; Bhat R. Bioactives from Agri-Food Wastes: Present Insights and Future Challenges. Molecules 2020, 25 (3), 510 10.3390/molecules25030510. PMC7037811 31991658

[ref42] Barba F. J.; Putnik P.; Bursać Kovačević D.; Poojary M. M.; Roohinejad S.; Lorenzo J. M.; Koubaa M. Impact of Conventional and Non-Conventional Processing on Prickly Pear (Opuntia Spp.) and Their Derived Products: From Preservation of Beverages to Valorization of by-Products. Trends Food Sci. Technol. 2017, 67, 260–270. 10.1016/j.tifs.2017.07.012.

[ref43] Le Bellec F.; Vaillant F.; Imbert E. Pitahaya (Hylocereus Spp.): A New Fruit Crop, a Market with a Future. Fruits 2006, 61 (4), 237–250. 10.1051/fruits:2006021.

[ref44] Sharma A.; Mazumdar B.; Keshav A. Valorization of Unsalable Amaranthus Tricolour Leaves by Microwave-Assisted Extraction of Betacyanin and Betaxanthin. Biomass Convers. Biorefinery 2021, 10.1007/s13399-020-01267-y.

[ref45] Maran J. P.; Priya B. Multivariate Statistical Analysis and Optimization of Ultrasound-Assisted Extraction of Natural Pigments from Waste Red Beet Stalks. J. Food Sci. Technol. 2016, 53 (1), 792–799. 10.1007/s13197-015-1988-8. 26788000 PMC4711424

[ref46] Nutter J.; Fernandez M. V.; Jagus R. J.; Agüero M. V. Development of an Aqueous Ultrasound-Assisted Extraction Process of Bioactive Compounds from Beet Leaves: A Proposal for Reducing Losses and Increasing Biomass Utilization. J. Sci. Food Agric. 2021, 101 (5), 1989–1997. 10.1002/jsfa.10815. 32914436

[ref47] Seremet D.; Durgo K.; Jokic S.; Huđek A.; Vojvodic Cebin A.; Mandura A.; Jurasovic J.; Komes D. Valorization of Banana and Red Beetroot Peels: Determination of Basic Macrocomponent Composition, Application of Novel Extraction Methodology and Assessment of Biological Activity in Vitro. Sustainability 2020, 12, 4539 10.3390/su12114539.

[ref48] Fernando G. S. N.; Wood K.; Papaioannou E. H.; Marshall L. J.; Sergeeva N. N.; Boesch C. Application of an Ultrasound-Assisted Extraction Method to Recover Betalains and Polyphenols from Red Beetroot Waste. ACS Sustain. Chem. Eng. 2021, 9 (26), 8736–8747. 10.1021/acssuschemeng.1c01203.

[ref49] Melgar B.; Dias M. I.; Barros L.; Ferreira I. C. F. R.; Rodriguez-Lopez A. D.; Garcia-Castello E. M. Ultrasound and Microwave Assisted Extraction of Opuntia Fruit Peels Biocompounds: Optimization and Comparison Using RSM-CCD. Molecules 2019, 24 (19), 3618 10.3390/molecules24193618. PMC6804160 31597259

[ref50] Koubaa M.; Barba F. J.; Grimi N.; Mhemdi H.; Koubaa W.; Boussetta N.; Vorobiev E. Recovery of Colorants from Red Prickly Pear Peels and Pulps Enhanced by Pulsed Electric Field and Ultrasound. Innov. Food Sci. Emerg. Technol. 2016, 37, 336–344. 10.1016/j.ifset.2016.04.015.

[ref51] Ferreres F.; Grosso C.; Gil-Izquierdo A.; Valentão P.; Mota A. T.; Andrade P. B. Optimization of the Recovery of High-Value Compounds from Pitaya Fruit by-Products Using Microwave-Assisted Extraction. Food Chem. 2017, 230, 463–474. 10.1016/j.foodchem.2017.03.061. 28407936

[ref52] Prieto-Santiago V.; Cavia M. M.; Alonso-Torre S. R.; Carrillo C. Relationship between Color and Betalain Content in Different Thermally Treated Beetroot Products. J. Food Sci. Technol. 2020, 57, 3305 10.1007/s13197-020-04363-z. 32728279 PMC7374684

[ref53] Dávila-Hernández G.; Sánchez-Pardo M. E.; Gutiérrez-López G. F.; Necoechea-Mondragon H.; Ortiz-Moreno A. Effect of Microwave Pretreatment on Bioactive Compounds Extraction from Xoconostle (Opuntia Joconostle) by-Products. Rev. Mex. Ing. Quim. 2018, 18 (1), 191–204. 10.24275/uam/izt/dcbi/revmexingquim/2019v18n1/Davila.

[ref54] Shen L.; Xiong X.; Zhang D.; Zekrumah M.; Hu Y.; Gu X.; Wang C.; Zou X. Optimization of Betacyanins from Agricultural By-Products Using Pressurized Hot Water Extraction for Antioxidant and in Vitro Oleic Acid-Induced Steatohepatitis Inhibitory Activity. J. Food Biochem. 2019, 43 (12), 1–14. 10.1111/jfbc.13044. 31515832

[ref55] Esatbeyoglu T.; Wagner A. E.; Schini-Kerth V. B.; Rimbach G. Betanin-A Food Colorant with Biological Activity. Mol. Nutr. Food Res. 2015, 59 (1), 36–47. 10.1002/mnfr.201400484. 25178819

[ref56] Gengatharan A.; Dykes G. A.; Choo W. S. Betalains: Natural Plant Pigments with Potential Application in Functional Foods. Lwt 2015, 64 (2), 645–649. 10.1016/j.lwt.2015.06.052.

[ref57] Gandía-Herrero F.; Escribano J.; García-Carmona F. Biological Activities of Plant Pigments Betalains. Crit. Rev. Food Sci. Nutr. 2016, 56 (6), 937–945. 10.1080/10408398.2012.740103. 25118005

[ref58] Fu Y.; Shi J.; Xie S. Y.; Zhang T. Y.; Soladoye O. P.; Aluko R. E. Red Beetroot Betalains: Perspectives on Extraction, Processing, and Potential Health Benefits. J. Agric. Food Chem. 2020, 68 (42), 11595–11611. 10.1021/acs.jafc.0c04241. 33040529

[ref59] Lechner J. F.; Stoner G. D. Red Beetroot and Betalains as Cancer Chemopreventative Agents. Molecules 2019, 24, 1602 10.3390/molecules24081602. PMC6515411 31018549

[ref60] Rahimi P.; Abedimanesh S.; Mesbah-Namin S. A.; Ostadrahimi A. Betalains, the Nature-Inspired Pigments, in Health and Diseases. Crit. Rev. Food Sci. Nutr. 2019, 59 (18), 2949–2978. 10.1080/10408398.2018.1479830. 29846082

[ref61] Rahimi P.; Mesbah-Namin S. A.; Ostadrahimi A.; Separham A.; Asghari Jafarabadi M. Betalain- and Betacyanin-Rich Supplements’ Impacts on the PBMC SIRT1 and LOX1 Genes Expression and Sirtuin-1 Protein Levels in Coronary Artery Disease Patients: A Pilot Crossover Clinical Trial. J. Funct. Foods 2019, 60, 103401 10.1016/j.jff.2019.06.003.

[ref62] Rahimi P.; Mesbah-Namin S. A.; Ostadrahimi A.; Abedimanesh S.; Separham A.; Asghary Jafarabadi M. Effects of Betalains on Atherogenic Risk Factors in Patients with Atherosclerotic Cardiovascular Disease. Food Funct. 2019, 10 (12), 8286–8297. 10.1039/C9FO02020A. 31723956

[ref63] Llorente B.; Martinez-Garcia J. F.; Stange C.; Rodriguez-Concepcion M. Illuminating Colors: Regulation of Carotenoid Biosynthesis and Accumulation by Light. Curr. Opin. Plant Biol. 2017, 37, 49–55. 10.1016/j.pbi.2017.03.011. 28411584

[ref64] Tian S. L.; Li L.; Shah S. N. M.; Gong Z. H. The Relationship between Red Fruit Colour Formation and Key Genes of Capsanthin Biosynthesis Pathway in Capsicum Annuum. Biol. Plant. 2015, 59, 507 10.1007/s10535-015-0529-7.

[ref65] Perrin F.; Hartmann L.; Dubois-Laurent C.; Welsch R.; Huet S.; Hamama L.; Briard M.; Peltier D.; Gagné S.; Geoffriau E. Carotenoid Gene Expression Explains the Difference of Carotenoid Accumulation in Carrot Root Tissues. Planta 2017, 245 (4), 737–747. 10.1007/s00425-016-2637-9. 27999990

[ref66] Gomez-Garcia M.; Ochoa-Alejo N. Biochemistry and Molecular Biology of Carotenoid Biosynthesis in Chili Peppers (Capsicum Spp.). International Journal of Molecular Sciences 2013, 14, 19025 10.3390/ijms140919025. 24065101 PMC3794819

[ref67] Llorente B.; Martinez-Garcia J. F.; Stange C.; Rodriguez-Concepcion M. Illuminating Colors: Regulation of Carotenoid Biosynthesis and Accumulation by Light. Current Opinion in Plant Biology 2017, 37, 49 10.1016/j.pbi.2017.03.011. 28411584

[ref68] Martínez-Zamora L.; Castillejo N.; Gómez P. A.; Artés-Hernández F.; Colla G.; De Pascale S. Amelioration Effect of LED Lighting in the Bioactive Compounds Synthesis during Carrot Sprouting. Agronomy 2021, 11, 304 10.3390/agronomy11020304.

[ref69] Martinez-Zamora L.; Castillejo N.; Artes-Hernandez F. Postharvest UV-B and Photoperiod with Blue + Red LEDs as Strategies to Stimulate Carotenogenesis in Bell Peppers. Appl. Sci. 2021, 11 (9), 3736 10.3390/app11093736.

[ref70] Dias M. G.; Borge G. I. A.; Kljak K.; Mandic A. I.; Mapelli-Brahm P.; Olmedilla-Alonso B.; Pintea A. M.; Ravasco F.; Tumbas Saponjac V.; Sereikaite J.; Vargas-Murga L.; Vulic J. J.; Melendez-Martinez A. J. European Database of Carotenoid Levels in Food. Foods 2021, 10 (5), 912 10.3390/foods10050912. 33919309 PMC8143354

[ref71] Kopsell D. A.; Sams C. E.; Barickman T. C.; Morrow R. C. Sprouting Broccoli Accumulate Higher Concentrations of Nutritionally Important Metabolites under Narrow-Band Light-Emitting Diode Lighting. J. Am. Soc. Hortic. Sci. 2014, 139, 469 10.21273/JASHS.139.4.469.

[ref72] Singh J.; Upadhyay A. K.; Prasad K.; Bahadur A.; Rai M. Variability of Carotenes, Vitamin C, E and Phenolics in Brassica Vegetables. J. Food Compos. Anal. 2007, 20 (2), 106–112. 10.1016/j.jfca.2006.08.002.

[ref73] Mohd Hassan N.; Yusof N. A.; Yahaya A. F.; Mohd Rozali N. N.; Othman R. Carotenoids of Capsicum Fruits: Pigment Profile and Health-Promoting Functional Attributes. Antioxidants 2019, 8, 469 10.3390/antiox8100469. PMC6827103 31600964

[ref74] Gupta P.; Sreelakshmi Y.; Sharma R. A Rapid and Sensitive Method for Determination of Carotenoids in Plant Tissues by High Performance Liquid Chromatography. Plant Methods 2015, 11, 5 10.1186/s13007-015-0051-0. 25688283 PMC4329677

[ref75] Raiola A.; Errico A.; Petruk G.; Monti D. M.; Barone A.; Rigano M. M. Bioactive Compounds in Brassicaceae Vegetables with a Role in the Prevention of Chronic Diseases. Molecules 2018, 23, 15 10.3390/molecules23010015. PMC5943923 29295478

[ref76] Trichopoulou A.; Martínez-González M. A.; Tong T. Y. N.; Forouhi N. G.; Khandelwal S.; Prabhakaran D.; Mozaffarian D.; de Lorgeril M. Definitions and Potential Health Benefits of the Mediterranean Diet: Views from Experts around the World. BMC Med. 2014, 12 (1), 112 10.1186/1741-7015-12-112. 25055810 PMC4222885

[ref77] González C. M.; Martínez L.; Ros G.; Nieto G. Evaluation of Nutritional Profile and Total Antioxidant Capacity of the Mediterranean Diet of Southern Spain. Food Sci. Nutr. 2019, 7 (12), 3853 10.1002/fsn3.1211. 31890163 PMC6924342

[ref78] Arscott S. A.; Tanumihardjo S. A. Carrots of Many Colors Provide Basic Nutrition and Bioavailable Phytochemicals Acting as a Functional Food. Compr. Rev. Food Sci. Food Saf. 2010, 9, 223 10.1111/j.1541-4337.2009.00103.x.

[ref79] Food and Agriculture Organization of the United Nations (FAO). The State of Food and Agriculture: Moving Forward on Food Loss and Waste Reduction; FAO: Rome, Italy, 2019.

[ref80] Nieto G.; Martínez L.; Castillo J.; Ros G. Effect of Hydroxytyrosol, Walnut and Olive Oil on Nutritional Profile of Low-Fat Chicken Frankfurters. Eur. J. Lipid Sci. Technol. 2017, 119 (9), 1600518 10.1002/ejlt.201600518.

[ref81] Wani F. A.; Rashid R.; Jabeen A.; Brochier B.; Yadav S.; Aijaz T.; Makroo H. A.; Dar B. N. Valorisation of Food Wastes to Produce Natural Pigments Using Non-Thermal Novel Extraction Methods: A Review. Int. J. Food Sci. Technol. 2021, 56, 4823 10.1111/ijfs.15267.

[ref82] Martínez-Hernández G. B.; Castillejo N.; Artés-Hernández F. Effect of Fresh-Cut Apples Fortification with Lycopene Microspheres, Revalorized from Tomato by-Products, during Shelf Life. Postharvest Biol. Technol. 2019, 156, 110925 10.1016/j.postharvbio.2019.05.026.

[ref83] Nagarajan J.; Pui Kay H.; Krishnamurthy N. P.; Ramakrishnan N. R.; Aldawoud T. M. S.; Galanakis C. M.; Wei O. C. Extraction of Carotenoids from Tomato Pomace via Water-Induced Hydrocolloidal Complexation. Biomolecules 2020, 10 (7), 1019 10.3390/biom10071019. PMC7407187 32660080

[ref84] Hooshmand H.; Shabanpour B.; Moosavi-Nasab M.; Golmakani M. T. Optimization of Carotenoids Extraction from Blue Crab (Portunus Pelagicus) and Shrimp (Penaeus Semisulcatus) Wastes Using Organic Solvents and Vegetable Oils. J. Food Process. Preserv. 2017, 41 (5), e13171 10.1111/jfpp.13171.

[ref85] Nour V.; Corbu A. R.; Rotaru P.; Karageorgou I.; Lalas S. Effect of Carotenoids, Extracted from Dry Tomato Waste, on the Stability and Characteristics of Various Vegetable Oils. Efecto de Los Carotenoides, Extraídos de Residuos de Tomates Secos, Sobre La Estabilidad y Características de Aceites Vegetales 2018, 69 (1), 238 10.3989/gya.0994171.

[ref86] Lasunon P.; Phonkerd N.; Tettawong P.; Sengkhamparn N. Effect of Microwave-Assisted Extraction on Bioactive Compounds from Industrial Tomato Waste and Its Antioxidant Activity. Food Res. 2021, 5 (2), 468–474. 10.26656/fr.2017.5(2).516.

[ref87] Goula A. M.; Ververi M.; Adamopoulou A.; Kaderides K. Green Ultrasound-Assisted Extraction of Carotenoids from Pomegranate Wastes Using Vegetable Oils. Ultrason. Sonochem. 2017, 34, 821–830. 10.1016/j.ultsonch.2016.07.022. 27773309

[ref88] Ajlouni S.; Premier R.; Tow W. W. Improving Extraction of Lycopene from Tomato Waste By-Products Using Ultrasonication and Freeze Drying. World J. Adv. Res. Rev. 2020, 05 (02), 177–185. 10.30574/wjarr.2020.5.2.0044.

[ref89] Luengo E.; Álvarez I.; Raso J. Improving Carotenoid Extraction from Tomato Waste by Pulsed Electric Fields. Front. Nutr. 2014, 10.3389/fnut.2014.00012. PMC4428368 25988115

[ref90] Pataro G.; Carullo D.; Falcone M.; Ferrari G. Recovery of Lycopene from Industrially Derived Tomato Processing By-Products by Pulsed Electric Fields-Assisted Extraction. Innov. Food Sci. Emerg. Technol. 2020, 63 (April), 102369 10.1016/j.ifset.2020.102369.

[ref91] Rubashvili I.; Tsitsagi M.; Ebralidze K.; Tsitsishvili V.; Eprikashvili L.; Chkhaidze M.; Zautashvili M. Extraction and Analysis of the Major Carotenoids of Agro-Industrial Waste Materials Using Sequential Extraction Techniques and High Performance Liquid Chromatography. Eurasian J. Anal. Chem. 2018, 13 ( (2), ), 10.29333/ejac/82931.

[ref92] de Andrade Lima M.; Kestekoglou I.; Charalampopoulos D.; Chatzifragkou A. Supercritical Fluid Extraction of Carotenoids from Vegetable Waste Matrices. Molecules 2019, 24 (3), 466 10.3390/molecules24030466. PMC6384789 30696092

[ref93] Kehili M.; Kammlott M.; Choura S.; Zammel A.; Zetzl C.; Smirnova I.; Allouche N.; Sayadi S. Supercritical CO2 Extraction and Antioxidant Activity of Lycopene and β-Carotene-Enriched Oleoresin from Tomato (Lycopersicum Esculentum L.) Peels by-Product of a Tunisian Industry. Food Bioprod. Process. 2017, 102, 340–349. 10.1016/j.fbp.2017.02.002.

[ref94] Pour Hosseini S. R.; Tavakoli O.; Sarrafzadeh M. H. Experimental Optimization of SC-CO2 Extraction of Carotenoids from Dunaliella Salina. J. Supercrit. Fluids 2017, 121, 89–95. 10.1016/j.supflu.2016.11.006.

[ref95] Dulf F. V.; Vodnar D. C.; Tosa M. I.; Dulf E. H. Simultaneous Enrichment of Grape Pomace with γ-Linolenic Acid and Carotenoids by Solid-State Fermentation with Zygomycetes Fungi and Antioxidant Potential of the Bioprocessed Substrates. Food Chem. 2020, 310, 125927 10.1016/j.foodchem.2019.125927. 31835232

[ref96] Grune T.; Lietz G.; Palou A.; Ross A. C.; Stahl W.; Tang G.; Thurnham D.; Yin S.-a.; Biesalski H. K. Beta-Carotene Is an Important Vitamin A Source for Humans. J. Nutr. 2010, 140 (12), 2268S 10.3945/jn.109.119024. 20980645 PMC3139236

[ref97] Fiedor J.; Burda K. Potential Role of Carotenoids as Antioxidants in Human Health and Disease. Nutrients 2014, 6 (2), 466–488. 10.3390/nu6020466. 24473231 PMC3942711

[ref98] Landrier J.-F.; Marcotorchino J.; Tourniaire F. Lipophilic Micronutrients and Adipose Tissue Biology. Nutrients 2012, 4 (11), 1622–1649. 10.3390/nu4111622. 23201837 PMC3509510

[ref99] Tourniaire F.; Gouranton E.; von Lintig J.; Keijer J.; Luisa Bonet M.; Amengual J.; Lietz G.; Landrier J.-F. β-Carotene Conversion Products and Their Effects on Adipose Tissue. Genes Nutr. 2009, 4 (3), 179 10.1007/s12263-009-0128-3. 19557453 PMC2745744

[ref100] Fiedor J.; Burda K. Potential Role of Carotenoids as Antioxidants in Human Health and Disease. Nutrients 2014, 6 (2), 466–488. 10.3390/nu6020466. 24473231 PMC3942711

[ref101] Gurmu F.; Hussein S.; Laing M. International Journal for Vitamin and Nutrition Research000194 2014, 84 (1–2), 65–78. 10.1024/0300-9831/a000194. 25835237

[ref102] Barker F. M.; Snodderly D. M.; Johnson E. J.; Schalch W.; Koepcke W.; Gerss J.; Neuringer M. Nutritional Manipulation of Primate Retinas, V: Effects of Lutein, Zeaxanthin, and n-3 Fatty Acids on Retinal Sensitivity to Blue-Light-Induced Damage. Invest. Ophthalmol. Vis. Sci. 2011, 52 (7), 3934–3942. 10.1167/iovs.10-5898. 21245404 PMC3175953

[ref103] Iwamoto T.; Hosoda K.; Hirano R.; Kurata H.; Matsumoto A.; Miki W.; Kamiyama M.; Itakura H.; Yamamoto S.; Kondo K. Inhibition of Low-Density Lipoprotein Oxidation by Astaxanthin. J. Atheroscler. Thromb. 2000, 7 (4), 216–222. 10.5551/jat1994.7.216. 11521685

[ref104] Yoshida H.; Yanai H.; Ito K.; Tomono Y.; Koikeda T.; Tsukahara H.; Tada N. Administration of Natural Astaxanthin Increases Serum HDL-Cholesterol and Adiponectin in Subjects with Mild Hyperlipidemia. Atherosclerosis 2010, 209 (2), 520–523. 10.1016/j.atherosclerosis.2009.10.012. 19892350

[ref105] Tanaka T.; Shnimizu M.; Moriwaki H. Cancer Chemoprevention by Carotenoids. Molecules 2012, 17 (3), 3202–3242. 10.3390/molecules17033202. 22418926 PMC6268471

[ref106] Keller J. N.; Schmitt F. A.; Scheff S. W.; Ding Q.; Chen Q.; Butterfield D. A.; Markesbery W. R. Evidence of Increased Oxidative Damage in Subjects with Mild Cognitive Impairment. Neurology 2005, 64 (7), 1152–1156. 10.1212/01.WNL.0000156156.13641.BA. 15824339

[ref107] Grodstein F.; Kang J. H.; Glynn R. J.; Cook N. R.; Gaziano J. M. A Randomized Trial of Beta Carotene Supplementation and Cognitive Function in Men: The Physicians’ Health Study II. Arch. Int. Med. 2007, 167 (20), 2184–2190. 10.1001/archinte.167.20.2184. 17998490

[ref108] Clinton S. K.; Emenhiser C.; Schwartz S. J.; Bostwick D. G.; Williams A. W.; Moore B. J.; Erdman J. W. Cis-Trans Lycopene Isomers, Carotenoids, and Retinol in the Human Prostate. Cancer Epidemiol. Biomarkers Prev. 1996, 5 (10), 823–833. 8896894

[ref109] Zu K.; Mucci L.; Rosner B. A.; Clinton S. K.; Loda M.; Stampfer M. J.; Giovannucci E. Dietary Lycopene, Angiogenesis, and Prostate Cancer: A Prospective Study in the Prostate-Specific Antigen Era. JNCI J. Natl. Cancer Inst. 2014, 106 (2), djt430 10.1093/jnci/djt430. 24463248 PMC3952200

[ref110] Rowles J. L.; Ranard K. M.; Smith J. W.; An R.; Erdman J. W. Increased Dietary and Circulating Lycopene Are Associated with Reduced Prostate Cancer Risk: A Systematic Review and Meta-Analysis. Prostate Cancer Prostatic Dis. 2017 204 2017, 20 (4), 361–377. 10.1038/pcan.2017.25. 28440323

[ref111] Erhardt A.; Stahl W.; Sies H.; Lirussi F.; Donner A.; Häussinger D. Plasma Levels of Vitamin E and Carotenoids Are Decreased in Patients with Nonalcoholic Steatohepatitis (NASH). Eur. J. Med. Res. 2011 162 2011, 16 (2), 76–78. 10.1186/2047-783X-16-2-76. PMC3353426 21463986

[ref112] Ip B. C.; Liu C.; Ausman L. M.; von Lintig J.; Wang X.-D. Lycopene Attenuated Hepatic Tumorigenesis via Differential Mechanisms Depending on Carotenoid Cleavage Enzyme in Mice. Cancer Prev. Res. (Phila). 2014, 7 (12), 1219–1227. 10.1158/1940-6207.CAPR-14-0154. 25293877 PMC4256117

[ref113] Böhm V. Lycopene and Heart Health. Mol. Nutr. Food Res. 2012, 56 (2), 296–303. 10.1002/mnfr.201100281. 22419532

[ref114] Müller L.; Caris-Veyrat C.; Lowe G.; Böhm V. Lycopene and Its Antioxidant Role in the Prevention of Cardiovascular Diseases–A Critical Review. Critical Reviews in Food Science and Nutrition 2016, 56 (11), 1868–1879. 10.1080/10408398.2013.801827. 25675359

[ref115] Krinsky N. I.; Johnson E. J. Carotenoid Actions and Their Relation to Health and Disease. Mol. Aspects Med. 2005, 26 (6), 459–516. 10.1016/j.mam.2005.10.001. 16309738

[ref116] Bays H. E. Adiposopathy Is “Sick Fat” a Cardiovascular Disease?. J. Am. Coll. Cardiol. 2011, 57 (25), 2461–2473. 10.1016/j.jacc.2011.02.038. 21679848

[ref117] Warnke I.; Goralczyk R.; Fuhrer E.; Schwager J. Dietary Constituents Reduce Lipid Accumulation in Murine C3H10 T1/2 Adipocytes: A Novel Fluorescent Method to Quantify Fat Droplets. Nutr. Metab. 2011, 8 (1), 30 10.1186/1743-7075-8-30. PMC3117678 21569430

[ref118] Luisa Bonet M.; Canas J. A.; Ribot J.; Palou A. Carotenoids and Their Conversion Products in the Control of Adipocyte Function, Adiposity and Obesity. Arch. Biochem. Biophys. 2015, 572, 112–125. 10.1016/j.abb.2015.02.022. 25721497

[ref119] Amengual J.; Gouranton E.; van Helden Y. G. J.; Hessel S.; Ribot J.; Kramer E.; Kiec-Wilk B.; Razny U.; Lietz G.; Wyss A.; Dembinska-Kiec A.; Palou A.; Keijer J.; Landrier J. F.; Bonet M. L.; von Lintig J. Beta-Carotene Reduces Body Adiposity of Mice via BCMO1. PLoS One 2011, 6 (6), e20644 10.1371/journal.pone.0020644. 21673813 PMC3106009

[ref120] Vishwanathan R.; Kuchan M. J.; Sen S.; Johnson E. J. Lutein and Preterm Infants with Decreased Concentrations of Brain Carotenoids. J. Pediatr. Gastroenterol. Nutr. 2014, 59 (5), 659–665. 10.1097/MPG.0000000000000389. 24691400

[ref121] Inanc A. L. Chlorophyll: Structural Properties, Health Benefits and Its Occurrence in Virgin Olive Oils. Akad. Gida 2009, 9 (2), 26–32.

[ref122] Ngamwonglumlert L.; Devahastin S.; Chiewchan N. Natural Colorants: Pigment Stability and Extraction Yield Enhancement via Utilization of Appropriate Pretreatment and Extraction Methods. Crit. Rev. Food Sci. Nutr. 2017, 57 (15), 3243–3259. 10.1080/10408398.2015.1109498. 26517806

[ref123] Humphrey A. M. Chlorophyll as a Color and Functional Ingredient. J. Food Sci. 2004, 69 (5), C422–C425. 10.1111/j.1365-2621.2004.tb10710.x.

[ref124] Derrien M.; Badr A.; Gosselin A.; Desjardins Y.; Angers P. Optimization of a Green Process for the Extraction of Lutein and Chlorophyll from Spinach By-Products Using Response Surface Methodology (RSM). LWT - Food Sci. Technol. 2017, 79, 170–177. 10.1016/j.lwt.2017.01.010.

[ref125] Zhang Z. H.; Wang L. H.; Zeng X. A.; Han Z.; Wang M. S. Effect of Pulsed Electric Fields (PEFs) on the Pigments Extracted from Spinach (Spinacia Oleracea L.). Innov. Food Sci. Emerg. Technol. 2017, 43, 26–34. 10.1016/j.ifset.2017.06.014.

[ref126] Chemat F.; Rombaut N.; Sicaire A. G.; Meullemiestre A.; Fabiano-Tixier A. S.; Abert-Vian M. Ultrasound Assisted Extraction of Food and Natural Products. Mechanisms, Techniques, Combinations, Protocols and Applications. A Review. Ultrason. Sonochem. 2017, 34, 540–560. 10.1016/j.ultsonch.2016.06.035. 27773280

[ref127] Agüero M. V.; Barg M. V.; Yommi A.; Camelo A.; Roura S. I. Postharvest Changes in Water Status and Chlorophyll Content of Lettuce (Lactuca Sativa L.) and Their Relationship with Overall Visual Quality. J. Food Sci. 2008, 73 (1), S47–S55. 10.1111/j.1750-3841.2007.00604.x. 18211369

[ref128] Kowalczyk K.; Sieczko L.; Goltsev V.; Kalaji H. M.; Gajc-Wolska J.; Gajewski M.; Gontar Ł.; Orliński P.; Niedzińska M.; Cetner M. D. Relationship between Chlorophyll FLuorescence Parameters and Quality of the Fresh and Stored Lettuce (Lactuca Sativa L.). Sci. Hortic. (Amsterdam). 2018, 235, 70–77. 10.1016/j.scienta.2018.02.054.

[ref129] Liu M.; Zhang L.; Ser S. L.; Cumming J. R.; Ku K. M. Comparative Phytonutrient Analysis of Broccoli By-Products: The Potentials for Broccoli by-Product Utilization. Molecules 2018, 23, 900 10.3390/molecules23040900. PMC6017511 29652847

[ref130] Ferreira S. S.; Monteiro F.; Passos C. P.; Silva A. M. S.; Wessel D. F.; Coimbra M. A.; Cardoso S. M. Blanching Impact on Pigments, Glucosinolates, and Phenolics of Dehydrated Broccoli by-Products. Food Res. Int. 2020, 132, 109055 10.1016/j.foodres.2020.109055. 32331656

[ref131] Borja-Martínez M.; Lozano-Sánchez J.; Borrás-Linares I.; Pedreño M. A.; Sabater-Jara A. B. Revalorization of Broccoli By-Products for Cosmetic Uses Using Supercritical Fluid Extraction. Antioxidants 2020, 9 (12), 1195 10.3390/antiox9121195. PMC7760773 33261112

[ref132] Zeyada N. N.; Zeitoum M. A. M.; Barbary O. M. Utilization of Some Vegetables and Fruits Waste As Natural Antioxidants. Alexandria J. Food Sci. Technol. 2008, 5 (1), 1–11. 10.21608/ajfs.2008.20136.

[ref133] Singh D. B.; Ahmed N.; Singh S. R.; Mir K. A.; Lal S. Variation in Chlorophyll and Carotenoid Contents in Kale (Brassica Oleracea) as Influenced by Cultivars and Harvesting Dates. Indian J. Agric. Sci. 2014, 84 (10), 1178–1181.

[ref134] Ruiz-Cano D.; Frutos M. J.; Hernández-Herrero J. A.; Pérez-Llamas F.; Zamora S. Effect of Chlorophyll Removal and Particle Size upon the Nutritional and Technological Properties of Powdered By-Products from Artichoke (Cynara Scolymus, L.) Industrial Canning. Int. J. Food Sci. Technol. 2015, 50 (11), 2383–2390. 10.1111/ijfs.12904.

[ref135] Fundo J. F.; Miller F. A.; Garcia E.; Santos J. R.; Silva C. L. M.; Brandão T. R. S. Physicochemical Characteristics, Bioactive Compounds and Antioxidant Activity in Juice, Pulp, Peel and Seeds of Cantaloupe Melon. J. Food Meas. Charact. 2018, 12 (1), 292–300. 10.1007/s11694-017-9640-0.

[ref136] Carbone K.; Amoriello T.; Iadecola R. Exploitation of Kiwi Juice Pomace for the Recovery of Natural Antioxidants through Microwave-Assisted Extraction. Agriculture 2020, 10 (10), 435 10.3390/agriculture10100435.

[ref137] Chaiareekitwat S.; Latif S.; Mahayothee B.; Khuwijitjaru P.; Nagle M.; Amawan S.; Müller J. Protein Composition, Chlorophyll, Carotenoids, and Cyanide Content of Cassava Leaves (Manihot Esculenta Crantz) as Influenced by Cultivar, Plant Age, and Leaf Position. Food Chem. 2022, 372, 131173 10.1016/j.foodchem.2021.131173. 34601424

[ref138] Kent M.; Welladsen H. M.; Mangott A.; Li Y. Nutritional Evaluation of Australian Microalgae as Potential Human Health Supplements. PLoS One 2015, 10 (2), e0118985 10.1371/journal.pone.0118985. 25723496 PMC4344213

[ref139] Aouir A.; Amiali M.; Bitam A.; Benchabane A.; Raghavan V. G. Comparison of the Biochemical Composition of Different Arthrospira Platensis Strains from Algeria, Chad and the USA. J. Food Meas. Charact. 2017, 11 (2), 913–923. 10.1007/s11694-016-9463-4.

[ref140] Tavanandi H. A.; Raghavarao K. S. M. S. Recovery of Chlorophylls from Spent Biomass of Arthrospira Platensis Obtained after Extraction of Phycobiliproteins. Bioresour. Technol. 2019, 271, 391–401. 10.1016/j.biortech.2018.09.141. 30296746

[ref141] Kong W.; Liu N.; Zhang J.; Yang Q.; Hua S.; Song H.; Xia C. Optimization of Ultrasound-Assisted Extraction Parameters of Chlorophyll from Chlorella Vulgaris Residue after Lipid Separation Using Response Surface Methodology. J. Food Sci. Technol. 2014, 51 (9), 2006–2013. 10.1007/s13197-012-0706-z. 25190857 PMC4152532

[ref142] Guo H.; Fang Z. Effect of Light Quality on the Cultivation of Chlorella Pyrenoidosa. E3S Web Conf. 2020, 143, 02033 10.1051/e3sconf/202014302033.

[ref143] Pagano I.; Campone L.; Celano R.; Piccinelli A. L.; Rastrelli L. Green Non-Conventional Techniques for the Extraction of Polyphenols from Agricultural Food by-Products: A Review. J. Chromatogr. A 2021, 1651, 462295 10.1016/j.chroma.2021.462295. 34118529

[ref144] Derrien M.; Aghabararnejad M.; Gosselin A.; Desjardins Y.; Angers P.; Boumghar Y. Optimization of Supercritical Carbon Dioxide Extraction of Lutein and Chlorophyll from Spinach By-Products Using Response Surface Methodology. Lwt 2018, 93, 79–87. 10.1016/j.lwt.2018.03.016.

[ref145] Cha K. H.; Lee H. J.; Koo S. Y.; Song D.-G.; Lee D.-U.; Pan C.-H. Optimization of Pressurized Liquid Extraction of Carotenoids and Chlorophylls from Chlorella Vulgaris. J. Agric. Food Chem. 2010, 58 (2), 793–797. 10.1021/jf902628j. 20028017

[ref146] Guo H.; Ma S.; Wang X.; Ren E.; Li Y. Microwave-Assisted Extraction of Chlorophyll from Filter Mud of Sugercane Mill and Component Analysis. Adv. Mater. Res. 2012, 518–523, 430–435. 10.4028/www.scientific.net/AMR.518-523.430.

[ref147] Michalak I.; Tuhy Ł.; Chojnacka K. Seaweed Extract by Microwave Assisted Extraction as Plant Growth Biostimulant. Open Chem. 2015, 13 (1), 1183–1195. 10.1515/chem-2015-0132.

[ref148] Nguyen N. H. K.; Diem An N. T.; Anh P. K.; Truc T. T. Microwave-Assisted Extraction of Chlorophyll and Polyphenol with Antioxidant Activity from Pandanus Amaryllifolius Roxb. in Vietnam. IOP Conf. Ser. Mater. Sci. Eng. 2021, 1166 (1), 012039 10.1088/1757-899X/1166/1/012039.

[ref149] Luengo E.; Condón-Abanto S.; Álvarez I.; Raso J. Effect of Pulsed Electric Field Treatments on Permeabilization and Extraction of Pigments from Chlorella Vulgaris. J. Membr. Biol. 2014, 247 (12), 1269–1277. 10.1007/s00232-014-9688-2. 24880235

[ref150] Pataro G.; Carullo D.; Ferrari G. PEF-Assisted Supercritical CO2 Extraction of Pigments from Microalgae Nannochloropsis Oceanica in a Continuous Flow System. Chem. Eng. Trans. 2019, 74, 97–102. 10.3303/CET1974017.

[ref151] Özkan G.; Ersus Bilek S. Enzyme-Assisted Extraction of Stabilized Chlorophyll from Spinach. Food Chem. 2015, 176, 152–157. 10.1016/j.foodchem.2014.12.059. 25624218

[ref152] Liu Y.; Perera C. O.; Suresh V. Comparison of Three Chosen Vegetables with Others from South East Asia for Their Lutein and Zeaxanthin Content. Food Chem. 2007, 101 (4), 1533–1539. 10.1016/j.foodchem.2006.04.005.

[ref153] da Silva Ferreira V.; Sant’Anna C. Impact of Culture Conditions on the Chlorophyll Content of Microalgae for Biotechnological Applications. World J. Microbiol. Biotechnol. 2017, 33 (1), 20 10.1007/s11274-016-2181-6. 27909993

[ref154] Lanfer-Marquez U. M.; Barros R. M. C.; Sinnecker P. Antioxidant Activity of Chlorophylls and Their Derivatives. Food Res. Int. 2005, 38 (8–9), 885–891. 10.1016/j.foodres.2005.02.012.

[ref155] Zhan R.; Wu J.; Ouyang J. In Vitro Antioxidant Activities of Sodium Zinc and Sodium Iron Chlorophyllins from Pine Needles. Food Technol. Biotechnol. 2014, 52 (4), 505–510. 10.17113/ftb.52.04.14.3592. 27904324 PMC5079145

[ref156] Kang Y. R.; Park J.; Jung S. K.; Chang Y. H. Synthesis, Characterization, and Functional Properties of Chlorophylls, Pheophytins, and Zn-Pheophytins. Food Chem. 2018, 245, 943–950. 10.1016/j.foodchem.2017.11.079. 29287463

[ref157] Hsu C.; Chao P.; Hu S.; Yang C. The Antioxidant and Free Radical Scavenging Activities of Chlorophylls and Pheophytins. Food Nutr. Sci. 2013, 4 (8A), 1–8. 10.4236/fns.2013.48A001.

[ref158] Cheng H. H.; Wang H. K.; Ito J.; Bastow K. F.; Tachibana Y.; Nakanishi Y.; Xu Z.; Luo T. Y.; Lee K. H. Cytotoxic Pheophorbide-Related Compounds from Clerodendrum Calamitosum and C. Cyrtophyllum. J. Nat. Prod. 2001, 64 (7), 915–919. 10.1021/np000595b. 11473423

[ref159] De Vogel J.; Jonker-Termont D. S. M. L.; Katan M. B.; Van Der Meer R. Natural Chlorophyll but Not Chlorophyllin Prevents Heme-Induced Cytotoxic and Hyperproliferative Effects in Rat Colon. J. Nutr. 2005, 135 (8), 1995–2000. 10.1093/jn/135.8.1995. 16046728

